# Fast and accurate quantification of insertion-site specific transgene levels from raw seed samples using solid-state nanopore technology

**DOI:** 10.1371/journal.pone.0226719

**Published:** 2019-12-27

**Authors:** Michael D. Pearson, Leslee Nguyen, Yanan Zhao, William L. McKenna, Trevor J. Morin, William B. Dunbar

**Affiliations:** Ontera, Inc., Santa Cruz, California, United States of America; University of Helsinki, FINLAND

## Abstract

Many modern crop varieties contain patented biotechnology traits, and an increasing number of these crops have multiple (stacked) traits. Fast and accurate determination of transgene levels is advantageous for a variety of use cases across the food, feed and fuel value chain. With the growing number of new transgenic crops, any technology used to quantify them should have robust assays that are simple to design and optimize, thereby facilitating the addition of new traits to an assay. Here we describe a PCR-based method that is simple to design, starts from whole seeds, and can be run to end-point in less than 5 minutes. Subsequent relative quantification (trait vs. non-trait) using capillary electrophoresis performed in 5% increments across the 0–100% range showed a mean absolute error of 1.9% (s.d. = 1.1%). We also show that the PCR assay can be coupled to non-optical solid-state nanopore sensors to give seed-to-trait quantification results with a mean absolute error of 2.3% (s.d. = 1.6%). In concert, the fast PCR and nanopore sensing stages demonstrated here can be fully integrated to produce seed-to-trait quantification results in less than 10 minutes, with high accuracy across the full dynamic range.

## Introduction

Over the last two decades, farmers around the globe have increasingly adopted the use of crops with genetic modifications that introduce novel traits, such as resistance to herbicides or pests. To address public concern around food safety, different countries have defined regulations that restrict these technologies and their use in food products, for example, which requires reliable detection and quantitative analytical methods for the implementation of labeling requirements [1]. Such methods are based either on DNA detection or protein detection. The presence or absence of a transgenic protein is commonly determined using lateral flow strips, or protein concentration is quantitatively estimated with an enzyme-linked immunosorbent assay (ELISA) in a laboratory setting [2]. DNA detection and quantification most often uses some version of Polymerase Chain Reaction (PCR), offering superior sensitivity, specificity and multiplexing power over protein assays [3]. In laboratory conditions, accurate relative quantification of trait vs. non-trait seeds from sample can be made using quantitative PCR (qPCR) or digital droplet PCR (ddPCR) [4–6]. Both techniques are relatively slow (30min to 3hr), however, and require purified DNA samples, fluorescent probes or dyes, and expensive sensitive optical devices. Isothermal amplification reactions can also semi-quantitatively assay for the transgene DNA in 20–30 minutes, but quantification performance is not as good as qPCR [7].

DNA assays traditionally used to accurately measure the relative amount of a transgene within a crop sample compare the measured copy number of the transgene to the measured copy number of a reference gene, which should be present in 100% of the plant genomes [4]. For this approach to give high accuracy, both of the independent measurements in the test must be accurate. For qPCR assays, this is only possible with purified DNA samples, highly efficient PCR primers and probes, and the reaction must be monitored at each cycle. For ddPCR, this is only possible with a lengthy PCR amplification and measurement. Typically, accuracy is optimized for the lower register of 0.1–5% of the quantification range [5], which is meaningful for the labeling requirements of regulations, for example, in the EU (0.9%), Taiwan (5%), and Japan (5%). While bias, defined as (calculated value–true value)/(true value) as a percentage, is an appropriate normalized measure of trueness in the lower register [5], we are focused on assessing the accuracy of our method across the full 0–100% dynamic range, in 5% increments, and therefore assess trueness by reporting the error (i.e., calculated value–true value), or absolute value of the error, without normalization. Otherwise, normalization has the effect of shrinking the bias as trait % increases, even if the error is plateaued. While qPCR methods in the literature produce up to 20% error within the upper register of the range, being optimized to discriminate 1.25-fold differences in trait abundance, we report a maximum of 5% for across the entire dynamic range.

In order to allow for a quantitative yet rapid test, we developed a novel variant of competitive PCR, which is a well-known and highly accurate method to quantify nucleic acid levels [8]. Standard competitive PCR relies on the addition of a precisely known amount of a synthetic DNA competitor to a reaction, that has the same two primer binding sites but a different length than the target, where the target is at an unknown level in the sample. When the PCR is performed, both DNA fragments are amplified using the same primers and components, which allows the amplification reactions to have close to identical kinetics. At the end point of the reaction, the relative abundance of the PCR products accurately reflects the relative amounts of the starting DNA templates.

To partially mimic the conditions of a competitive PCR assay, we developed a new assay method where the two amplified DNA molecules have an identical sequence on one of their ends but not the other. This arrangement can be found at the genomic location of a transgene insertion, when compared to its associated wildtype variant. In a sample containing both the transgene-inserted and the wildtype templates, an end-point PCR is then performed to generate both amplicons in the same sample using three primers, one of which is common to both amplifications. Because the common primer is consumed at a higher rate than either of the other primers, it becomes a limiting reagent that therefore holds both products at close to the same amplification rate. At endpoint, the ratio of the two products is reproducibly correlated to the ratio of the starting template. Since both amplifications occur in the same tube, any inhibition due to crude DNA extractions affects both reactions at the same level, and thus has a minimal effect on the ratio of the products.

By designing primers to give different sized PCR amplicons, the end-point ratio can be determined by any method able to separate and quantify them. The most common laboratory methods for this are gel electrophoresis or capillary electrophoresis, with quantification by using a fluorescent intercalating dye or UV absorbance. Another method is to not separate the PCR products at all, and simply quantify their relative amounts by recording the change in electrical signal when individual DNA molecules translocate through a solid-state nanopore sensor [9].

Briefly, a solid-state nanopore is a nanoscale hole formed in a thin solid-state membrane that separates two aqueous volumes [10,11]. An amplifier applies a voltage across the membrane while measuring the ionic current through the open pore. When a single charged molecule such as a double-stranded DNA is captured and driven through the pore by electrophoresis, the measured current shifts, and the shift depth and duration properties are used to characterize each single-molecule “event.” After recording 100–1000 events in a few minutes, the event distributions are analyzed to characterize the corresponding molecules present [12]. Nanopore sensing thus offers a simple and high-throughput electrical read-out, with an instrument that can have a small footprint at low cost [9]. Prior research has shown that nanopores can discriminate DNA by length, since longer DNA produce longer duration events [11,13]. For example, length-based discrimination with Bayesian classification has been used for molecular “fingerprinting” in a diagnostic application [14]. We proposed a nanopore-based method for relative quantification of two DNA populations [15], which is applied here using length-based discrimination but is compatible with any other nanopore-based scheme for DNA discrimination [16,17].

To demonstrate a use case, we chose to validate the method by quantification of the relative weight of soybeans that comprise the GTS40-3-2 event, which confers resistance to glyphosate [1], from a 35-gram mixture of seeds. To show the simplicity of assay design and robustness of the method, we designed three different 3-primer assays, and demonstrate insertion-site specific quantification. We also demonstrate that the method works with crude samples, and that the PCR can be performed in under five minutes. Lastly, we show that the post-PCR ratio can be accurately and efficiently quantified using solid-state nanopores.

## Materials and methods

### Three-primer assay design and quantification

The method is presented as a recipe over the following sections, and we note that alternative DNA extraction and PCR protocols are compatible with the method. As a proof-of-concept example, soybeans that comprise the GTS40-3-2 event (Trait Seeds) and conventional soybeans (Non-Trait Seeds) are used to make mixtures for relative quantification. These mixtures are defined by the amount of Trait Seed material in the mix (%Trait), with 0%Trait having only Non-Trait Seed and 100%Trait having only Trait Seed.

#### 1. Obtain DNA sequences

We first obtain genomic DNA sequence for one of the junctions where the transgene of interest was inserted into the genome (the Trait DNA). About 400 base pairs on either side of the junction are needed. The same length of corresponding genomic sequence from the non-transgenic organism found in the mixture is also needed (the Non-Trait DNA). Half of the two sequences should be identical, or nearly identical (the Common DNA). The procedure we used to obtain the Trait DNA and Non-Trait DNA are found in the Genomic DNA Sequences Protocol.

#### 2. Design the three primers

Using the PCR Primer Design Protocol with the Trait DNA, we next designed two oligonucleotide PCR primers that generate a PCR amplicon 80–400 base pairs in length (the Trait PCR) that crosses the junction in the Trait DNA. There should be one primer that binds within the transgene (the Trait Primer), and a second primer that binds to the Common DNA (the Common Primer). Using the PCR Primer Design Protocol with the Non-Trait DNA, we use the Common Primer to design another PCR primer (the Non-Trait Primer) that crosses the site that was disrupted when the transgene was inserted. The amplicon generated by these two primers (the Non-Trait PCR) should have a length that is sufficiently different from the Trait PCR to facilitate relative quantification of Trait vs. Non-Trait amplicons following end-point PCR. Nominally, the difference in length should be at least 100 bp for facile quantification using either capillary electrophoresis or nanopore measurement. All three primers (Trait, Non-Trait, Common) together make an assay. To demonstrate diversity of primer design, we made sixteen different assays shown in S1 Table for the model Trait vs. Non-Trait system, three of which (assays 2, 14 and 16) were selected to showcase the full method presented here.

#### 3. Produce reference DNA templates

Seed mixtures: Reference DNA templates of 0%Trait and 100%Trait seeds are produced, as well as one from a 50%Trait mixture of seeds. In our example, we have used the Quick DNA Extraction Protocol to make crude extracts from whole soybeans in less than one minute. The resulting 0%, 50% and 100% extracts from seeds are denoted as “**%Trait-Extract**” in figures and tables.

Extract mixtures: To produce accurate mixtures that combine the 0% and 100% extracts, the extracts are normalized to the same A260 absorbance. The 0%Trait and 100%Trait extracts were then mixed by volume to make a total set of 19 additional extracts, from 5%Trait to 95%Trait, in 5%Trait increments. These extracts are denoted as “**%Trait-Extract-Mix**” in figures and tables.

#### 4. Test the assay for specificity

**Fig 1:**
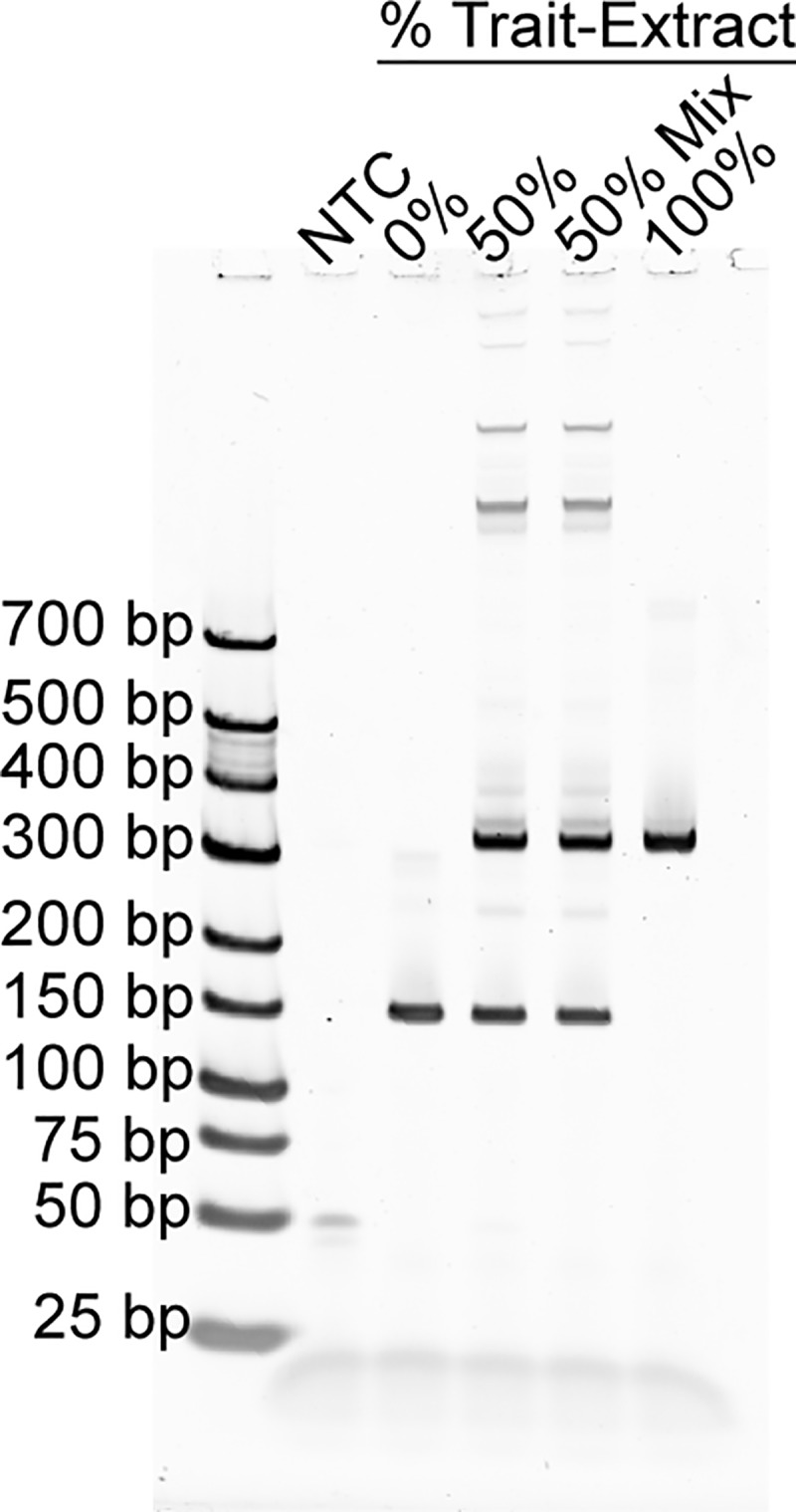
A specificity test for assay 2 qualitatively shows the expected ratios of Trait vs. Non-Trait amplicons. The %Trait-Extract PCR products are made from 0%Trait, 50%Trait, and 100%Trait seed mixes, with the exception of the 50% Mix which was made from Extracts (Step 3). The products are visualized using the Gel Electrophoresis Protocol, showing the Trait 298 bp and Non-trait 153 bp amplicon lengths.

Assays should be checked for specificity with the 0%, 50%, and 100%Trait-Extracts, as well as the 50%Trait-Extract-Mix. Using PCR Protocol A, all sixteen assays were tested for specificity (S1 Fig). Successful assays should have single PCR amplicons for 0%Trait PCR and 100%Trait PCR, while both amplicons (Trait PCR and Non-Trait PCR) should be present at similar levels for the 50%Trait. The PCRs can be qualitatively visualized using the Gel Electrophoresis Protocol, as shown for assay 2 in Fig 1, and shown also for assays 14 and 16 in S2 Fig. When visualized on a gel, the PCRs containing both templates often have bands higher up on the gel, which is likely the result of hetero-duplex formation but does not have a negative impact on the results. The PCR shown in Fig 1 was next quantitated using the Capillary Electrophoresis Protocol. The quantification was reported as **“%Trait PCR”**, which is the percentage of Trait PCR (in ng) to total PCR (Trait PCR and Non-Trait PCR in ng). The %Trait PCR of the 50%Trait-Extract and 50%Trait-Extract-Mix showed close to the same value for assay 2 (Table 1), and also for assays 14 and 16 (S2 Tables). Quantification of 50%Trait-Extract was also tested for all sixteen assays, with assays 2 and 14 showing values within 10% of the true 50% value (S2 Table). While we used Capillary Electrophoresis to quantitate the reactions in these examples, any method that can quantify the relative amount of the two amplicons may be used.

**Table 1:** PCR quantification of Fig 1 data using the capillary electrophoresis protocol.

	%Trait PCR
**0%Trait-Extract**	0.0%
**50%Trait-Extract**	51.0%
**50%Trait-Extract-Mix**	47.8%
**100%Trait-Extract**	100.0%

#### 5. Generate a reference data set

A reference data set is next created and used to make a Calibration Equation. The reference data can be generated from any amount of test PCRs. The minimum number of test PCRs is a single reaction with the 50%Trait-Extract-Mix. The accuracy of the assays will generally improve with additional reference reactions. For our example, we used the twenty-one %Trait-Extract-Mix reactions (0-to-100%, in 5% increments), and using PCR Protocol A with assay 2. These PCRs were performed in two sets (Experiment A and Experiment B), but as long as the same protocol is used, they could be performed all together, or divided into smaller subsets. The PCRs were qualitatively analyzed using the Gel Electrophoresis Protocol (S3 Fig), and quantitatively analyzed using the Capillary Electrophoresis Protocol to yield a set of %Trait PCR values (Table 2).

**Table 2:** The %Trait PCR values produced using the capillary electrophoresis protocol for reference experiments A and B.

Assay 2 Experiment A		Assay 2 Experiment B	
%Trait-Extract-Mix	%Trait PCR	%Trait-Extract-Mix	%Trait PCR
**0%**	0.0%	**0%**	0.0%
**10%**	13.6%	**5%**	5.2%
**20%**	17.4%	**15%**	17.1%
**30%**	27.0%	**25%**	23.5%
**40%**	34.0%	**35%**	34.1%
**50%**	45.7%	**45%**	47.1%
**60%**	54.1%	**50%**	46.8%
**70%**	65.0%	**55%**	47.5%
**80%**	71.2%	**65%**	57.3%
**90%**	85.9%	**75%**	67.2%
**100%**	100.0%	**85%**	81.3%
		**95%**	93.0%
		**100%**	100.0%

#### 6. Generate a calibration equation

**Fig 2:**
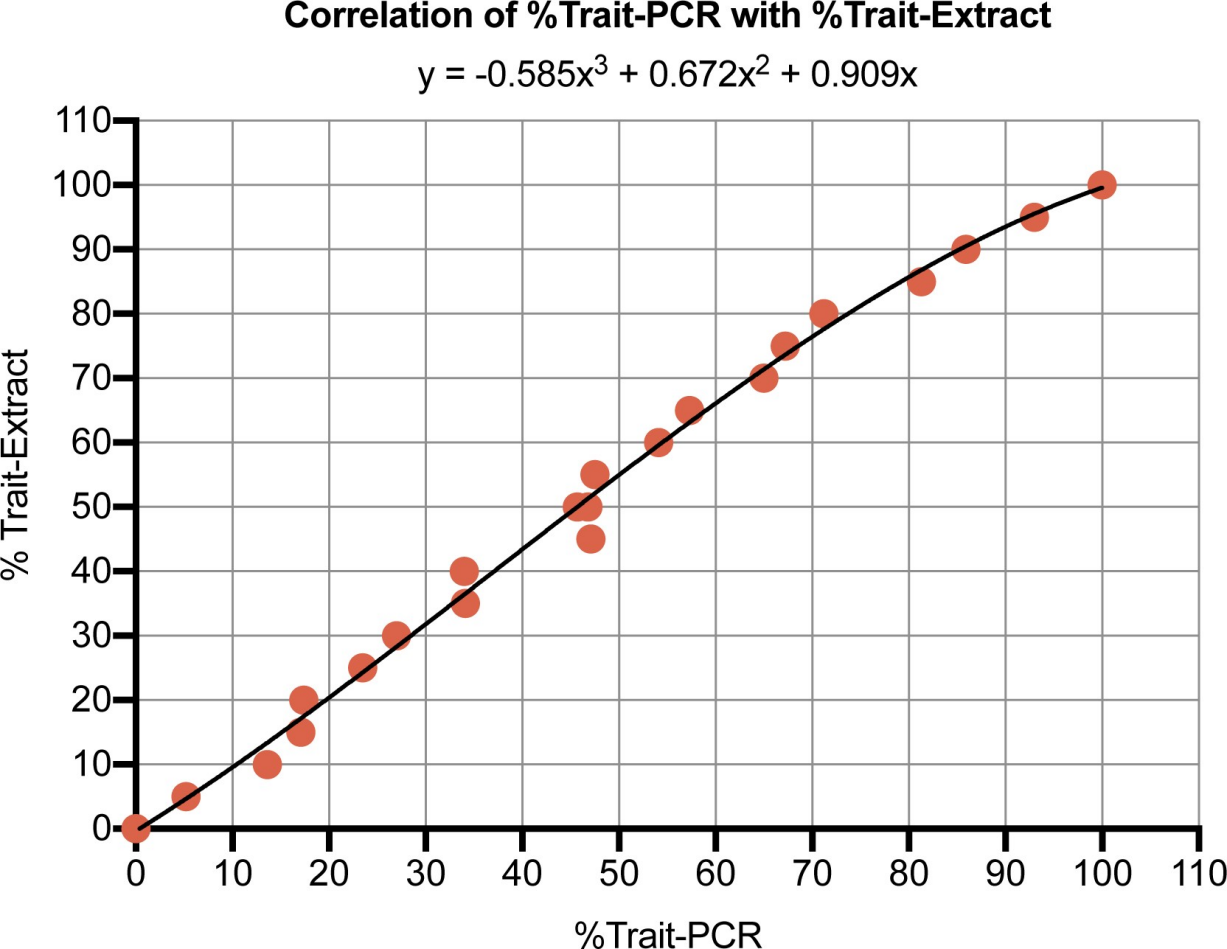
The correlation between %Trait-Extract and %Trait PCR is used to fit a Calibration Equation. The 21 data points are the combined values from Experiments A and B in Table 2. %Trait PCR values are plotted on the horizontal axis so the fit can convert %Trait PCR values, produced form analysis of unknown raw seed mixtures, into %Trait-Extract predictions.

The %Trait PCR values are next plotted vs the %Trait-Extract-Mix values. We used Microsoft Excel Software to plot the %Trait PCR values on the X-axis, and the input %Trait-Extract-Mix value on the Y-axis. The software was then used to perform regression analysis to generate a 3^rd^ degree Calibration Equation using all 21 data points combining Experiments A and B (Fig 2). To demonstrate that this can be done with fewer points, a fit was also made using only the 50%Trait PCR (and anchored at (0,0) and (100,100)) to generate a 2^nd^ degree Calibration Equation. For the assay 2 reference data, for example, the equation produced using only the 50%Trait PCR was: y = -0.151x^2^ + 1.151x, where x is %Trait PCR and y is %Trait. The 3^rd^ degree and 2^nd^ degree Calibration Equations calculated for assays 2, 14 and 16 using Experiment A and B data are reported in S3A–S3I Tables. Note that by having %Trait PCR values on the X-axis, test data produced from raw seed mixtures can be analyzed (as described in the next section) to produce a %Trait PCR value as the X value, and the equation can be directly applied to produce a %Trait-Extract estimate as the Y value output.

#### 7. Produce test DNA templates

Test DNA templates of mixed Trait and Non-Trait organisms were produced next. For our example, we weighed out 21 mixes of whole soybeans, and used the Quick Extraction Protocol on each to make 21 different %Trait extracts, from 0%Trait to 100%Trait in 5%Trait increments. The test extracts were not normalized to a certain A260 reading, in part to emulate the condition of testing from crude seed-mixture extracts. These test extracts are noted as “**%Trait-Extract**” in figures and tables.

#### 8. Generate test data

Using exactly the same protocols used to produce the %Trait PCR values in the reference data sets, test samples can be used to produce test %Trait PCR values. In our example, the 21 test DNA templates made in step 7 were used with assay 2 and PCR Protocol A to create a test set with 21 test reactions (termed “Experiment C”). The test set was qualitatively analyzed with the Gel Electrophoresis Protocol (S4 Fig), and quantitatively analyzed with the Capillary Electrophoresis Protocol to produce a set of test %Trait PCR values (Table 3).

**Table 3:** The %Trait PCR values produced using the capillary electrophoresis protocol for test experiment C.

Assay 2 Experiment C	
%Trait-Extract	%Trait PCR
**0%**	0.0%
**5%**	3.0%
**10%**	8.7%
**15%**	16.5%
**20%**	18.5%
**25%**	23.1%
**30%**	29.0%
**35%**	31.2%
**40%**	37.7%
**45%**	38.5%
**50%**	47.6%
**55%**	47.6%
**60%**	50.5%
**65%**	58.2%
**70%**	63.0%
**75%**	67.1%
**80%**	71.6%
**85%**	75.2%
**90%**	84.2%
**95%**	96.3%
**100%**	100.0%

#### 9. Calculation of %Trait-Extract from %Trait PCR

Using the calibration equations generated in step 7, the %Trait-Extract values can be estimated from the %Trait PCR values. For our example, we calculated the %Trait-Extract values from each of the 21 tests of Experiment C using the calibration equations that were derived from Experiments A and B data. When using the 3^rd^ degree equation, the average absolute error between the true %Trait-Extract and the calculated value was 1.87%, with the largest error of -4.47%. Using the 2^nd^ degree equation, the average absolute error was 2.82%, with no individual difference of more than 7% (Table 4). The mean and standard deviation of the absolute error values reported at the bottom of Table 4 excluded the 0% and 100% error values, since there corrected values had nearly zero error by design of the calibration method.

**Table 4:** Calculated %Trait-Extract values by applying the calibration equations to the test %Trait PCR data from Table 3.

Assay 2 Experiment C				
%Trait-Extract	3rd Degree Equation		2nd Degree Equation	
	Calculated %Trait	Error (Calc.–True)	Calculated %Trait	Error (Calc.–True)
**0%**	0.00%	0.00%	0.00%	0.00%
**5%**	2.77%	-2.23%	3.44%	-1.56%
**10%**	8.35%	-1.65%	9.90%	-0.10%
**15%**	16.53%	1.53%	18.58%	3.58%
**20%**	18.71%	-1.29%	20.77%	0.77%
**25%**	23.83%	-1.17%	25.78%	0.78%
**30%**	30.56%	0.56%	32.11%	2.11%
**35%**	33.11%	-1.89%	34.44%	-0.56%
**40%**	40.68%	0.68%	41.24%	1.24%
**45%**	41.62%	-3.38%	42.07%	-2.93%
**50%**	52.20%	2.20%	51.36%	1.36%
**55%**	52.20%	-2.80%	51.36%	-3.64%
**60%**	55.53%	-4.47%	54.27%	-5.73%
**65%**	64.15%	-0.85%	61.87%	-3.13%
**70%**	69.33%	-0.67%	66.51%	-3.49%
**75%**	73.58%	-1.42%	70.43%	-4.57%
**80%**	78.05%	-1.95%	74.67%	-5.33%
**85%**	81.45%	-3.55%	78.01%	-6.99%
**90%**	89.16%	-0.84%	86.20%	-3.80%
**95%**	97.36%	2.36%	96.83%	1.83%
**100%**	99.28%	-0.72%	99.99%	-0.01%
**Average |Error|**^**a**^		1.87%		2.82%
**Standard Deviation |Error|**^**a**^		1.05%		1.87%

^a^The average and standard deviation of the absolute error exclude 0% and 100% data.

### Protocols

#### Soybean varieties protocol

All assays were tested using either soybeans (Trait soybeans) containing the GTS40-3-2 event that confers glyphosate tolerance (a blend of Big Fellow, Large Lad, and Whitetail Thicket), a non-transgenic heirloom ‘Black Jet’ soybean (Non-Trait soybeans), or a defined weight/weight mixture (%Trait) of the two soybean varieties.

#### Genomic DNA sequences protocol

The DNA sequences (S4 Table) at the junctions of the transgenic insertion in Trait soybeans were taken from the literature [18]. As the transgene was integrated into the genome with a complex rearrangement, the two ends (Junction A, and Junction B) are connected to two genomic locations that are not contiguous in conventional soybeans. The DNA sequences of the two corresponding insertion sites in conventional soybeans (Non-Trait) were obtained with a BLAST search of the database of the Legume Information System (https://legumeinfo.org/home) using the transgene junction sequences.

#### Primer design protocol

Three 3-primer sets of oligonucleotide primers (assays 2, 14 and 16 in S1 Table) were designed using PrimerQuest software (Integrated DNA Technologies https://www.idtdna.com/PrimerQuest/) with the following parameters: Tm 59ºC-65ºC, GC content 35%-65%, length 17nt-30nt. In order to design 3-primer sets, first 2-primer sets were designed to give Non-Trait amplification products between 75nt and 400nt in length, that include the DNA sequence that was disrupted when the transgene was integrated into the chromosome. Primers used to amplify these Non-Trait fragments were screened to avoid all known DNA variants using the Soybean Genome Variation Map (BIGD http://bigd.big.ac.cn/gvm/search). Each of the acceptable primers was then individually used as a starting point to design a primer that would amplify only Trait DNA, and not Non-Trait DNA. Because the transgene insertion event is not a simple insertion, both Junction A and Junction B were chosen to design assays. The amplification product lengths were chosen to make pairs of DNA amplicons (one amplified only from Non-Trait soybeans, and one amplified only from Trait soybeans) differing by at least 100bp for facile quantification.

#### DNA extraction protocol

35 grams of soybeans (~250 seeds) were ground with an Oster blender on the highest setting for 10 seconds, and 100 mL of 250 mM NaOH was added to the ground seeds and shaken by hand for 15 seconds. Roughly 75% of the contents in the cup were poured into an Aeropress coffee maker and filtered with a paper filter, yielding 10–15 mL of filtered extract. Using a blunt p1000 pipette tip, 1 mL of filtered extract was diluted 1:10 by adding 9 mL of water. The mixture was vortexed for 10 seconds, then another 1:10 serial dilution was performed in 5 mL final volume, for a final dilution of 1:100. The 1:100 diluted extract was then aliquoted and frozen at -20ºC, diluted further, or directly used as the template for the PCR reaction.

#### PCR protocol A

4 μL of a 1:1000 dilution from a Quick DNA Extraction was added to 21 μL of PCR master mix (5 μL 10x HF buffer (New England Biolabs), 0.5 μL Phusion Hot Start Flex DNA Polymerase (New England Biolabs), 0.5 μL 10 mM dNTPs, 0.5 μL 30 μM each of 3 oligonucleotide primers (Integrated DNA Technologies), 13.5 μL water for a final volume of 25μL). The amplifications (in triplicate) were performed using a C1000 Touch thermocycler (Bio-Rad) [95ºC for 30 sec, followed by 35 cycles of 95ºC for 5 sec, 60ºC 10 sec, 72ºC 10 sec, followed by 72ºC for 30 sec]. The triplicate amplification reactions were then merged to a final volume of 75 μL before analysis.

#### PCR protocol B

2 μL of a 1:100 dilution of each of extract was added to 10.5 μL of PCR master mix (2.5 μL 10x HF buffer (New England Biolabs), 1.5 μL Phusion Hot Start Flex DNA Polymerase (New England Biolabs), 0.25 μL 50 mM MgCl2, 0.25μL 10mM dNTPs, 0.75 μL of Non-trait specific and 1 μL of Trait specific and Common oligonucleotide primers at 100 μM (Integrated DNA Technologies), 3.25μL water for a final volume of 12.5μL). 12.5μL amplifications (in triplicate) were performed using a NextGenPCR thermocycler (Molecular Biology Systems (MBS)) [98ºC for 5sec, followed by 35 cycles of 98ºC for 1sec, 55ºC 1sec, 75ºC 3sec, for a total run time of 4min 52sec]. The triplicate amplification reactions were then merged to a final volume of ~37μL.

#### Gel electrophoresis protocol

3 μL of each of the merged PCR products was analyzed using a 6% TBE PAGE gel run at 200V for 25 minutes, followed by staining with SybrGreen for 15 minutes and visualization with a Bio-Rad ChemiDoc MP.

#### Capillary electrophoresis protocol

2 μL of a PCR reaction was quantitatively analyzed using a Fragment Analyzer System (Agilent) and a dsDNA 910 reagent kit. The percentage of the total ng (Trait and Non-Trait) that was contained in the trait specific amplicon was recorded as %Trait PCR, and used for analysis.

#### Nanopore protocol

PCR reactions were diluted 1 to 50 into a nanopore recording buffer, which comprised of 4.0 M LiCl, 50 mM Tris HCl pH 8.8, 5 mM EDTA, and 10% PEG 200 v/v. Nanopore chip fabrication and the injection molded test strip used to package and fluidically seal a chip are described in S1 Text. For measuring a sample, approximately 10 μL of diluted sample was pipetted into the test strip and 100 mV bias was applied to the nanopore chip (trans side positive) using a prototype voltage-clamp amplifier [9]. Ionic current data was recorded using custom software at a sampling rate of 125 kHz for approximately 5 minutes, or enough time to collect ~1000 molecular translocation events for each reagent. Each sample %Trait-Extract was recorded on 4 independent pores. Nanopore diameters ranged in size from 25–41 nm across all data sets (pore size range is discussed in S1 Text, and size details per nanopore device are reported in S6 Table). Control datasets, for model training and quantification correction, were collected for each pore just prior to each test data, as described in S2 Text. (This subject matter is related to PCT Application No. PCT/US2019/050087, unpublished).

## Results

Having completed the presentation of the method through a working example with representative data, we can make some general comments here. First, observe that the workflow would be simplified without using a reference-data-derived calibration, in which case the %Trait PCR values can provide direct estimates for the %Trait values. However, this is implicitly equivalent to assuming a calibration equation equal to a straight line through (0,0) with slope 1, which generally produces higher errors. For the data in Table 3, for example, the mean absolute error (excluding 0% and 100%) is 4.64% (s.d. 3.07%), which is clearly inferior to the results using reference data to derive the calibration equations.

Across the entire dynamic range, the absolute error with 3^rd^ degree calibration equation has the mean value 1.87% and standard deviation 1.05% across 19 error values, which corresponds to 0.24% standard error of and a 95% confidence interval of 1.39% to 2.34% (mean ± 0.47%). To test variability, we repeated Experiment C data two more times (Experiments C1-C3, S5 Fig), and all three sets of results produced consistent results. Moreover, by averaging across the three sets, the error was further reduced (S5 Table). Specifically, the triplicate-average of the mean absolute error (excluding 0% and 100%) is 3.86% (s.d. 2.63%) without calibration, and 1.08% (s.d. 0.86%) and 1.94% (s.d. 1.30%) with 3^rd^ degree and 2^nd^ degree calibration, respectively. The triplicate-averages had a mean standard deviation of 1.6%.

### Using solid-state nanopores to quantify %Trait

To show that the method outlined above is not limited to a particular method of quantification, we also demonstrate the use of nanopore technology for measuring and calculating the %Trait using the Experiment C samples. The nanopore-based trait quantification method is described in detail in [15] and S2 Text, with relevant portions described here.

Using the Nanopore Protocol, sets of four independent nanopores were used to measure and quantitate each %Trait-Extract from the Experiment C samples. Prior to running %Trait-Extract samples, three controls are sequentially run on each nanopore: 0%Trait-Extract, 50%Trait-Extract, and 100% Trait-Extract. The controls are used to build a support vector machine (SVM)-based model for assignment of trait events vs. non-trait events, and also to compensate for a difference in the nanopore capture frequency of the two different length amplicons 2 (Trait 298 bp, Non-trait 153 bp). While the %Trait-Extract values were used as internal controls for nanopore quantification, the resulting %Trait PCR estimates can be subsequently calibrated using the calibration equations derived from the %Trait-Extract-Mix reference data (Experiments A and B). Notably, those calibrations were derived using capillary electrophoresis results, and a calibration based on nanopore-analyzed reference data could further improve accuracy, though this was not explored. As with the capillary electrophoresis results for Experiment C reagents, the quadruplet nanopore measurements were generated for 0% to 100% in 5%-increments (21 values).

The results of applying the SVM method to quadruplet nanopore reads are shown in Table 5. Each of the reported %Trait PCR values are the average of the four values generated with four separate nanopores (S6 Table). As with the capillary electrophoresis results for Experiment C, the %Trait PCR estimates consistently under predicted the %Trait-Extract value (Table 3), and quantification improved for both methods (CE, nanopore) by using calibration (Tables 4 and 5). Using the SVM method, the largest difference between %Trait-Extract and %-Trait PCR was -7.14% and the average absolute error was 3.69% (s.d. 2.17%). The spread of each SVM prediction (defined as the standard deviation of the mean) has an average value of 2.87%. The 2^nd^ degree calibration equation derived for assay 2 using reference mixtures (Experiments A and B) was applied to the SVM data, which reduced the average absolute error to 2.29% (s.d. 1.58%) and lowered the maximum deviation to -5%.

**Table 5:** %Trait PCR predictions generated by applying the support vector machine method to nanopore data and also the 2^nd^ degree calibration equation.

**Nanopore Quantification of Assay 2 Experiment C**				
%Trait-Extract	SVM Prediction		2nd Degree Equation	
	%Trait PCR	Error (Calc.–True)	Calculated %Trait	Error (Calc.–True)
**5%**	0.00%	-5.00%	0.00%	-5.00%
**10%**	5.32%	-4.68%	6.08%	-3.92%
**15%**	13.90%	-1.10%	15.70%	0.70%
**20%**	12.87%	-7.14%	14.56%	-5.44%
**25%**	18.83%	-6.17%	21.13%	-3.87%
**30%**	28.86%	-1.15%	31.95%	1.95%
**35%**	34.92%	-0.09%	38.34%	3.34%
**40%**	35.31%	-4.69%	38.75%	-1.25%
**45%**	44.48%	-0.52%	48.20%	3.20%
**50%**	46.54%	-3.47%	50.29%	0.29%
**55%**	48.29%	-6.71%	52.06%	-2.94%
**60%**	55.23%	-4.77%	58.96%	-1.04%
**65%**	59.83%	-5.17%	63.46%	-1.54%
**70%**	67.53%	-2.47%	70.84%	0.84%
**75%**	69.87%	-5.13%	73.05%	-1.95%
**80%**	77.15%	-2.86%	79.80%	-0.20%
**85%**	83.94%	-1.06%	85.97%	0.97%
**90%**	83.99%	-6.01%	86.02%	-3.98%
**95%**	93.01%	-1.99%	93.98%	-1.02%
**Average |Error|**		3.69%		2.29%
**Standard Deviation |Error|**		2.17%		1.58%

To shorten the total assay time, we reduced the three-control workflow required by the SVM method, and developed a single-control method based on principle component analysis (PCA)

(S2 Text). The PCA method only requires a 50%Trait-Extract to be run prior to the %Trait-Extract to be quantified. The method was applied to a subset of the same data used with SVM analysis, by removing the 0%Trait-Extract and 100% Trait-Extract data and using only the 50%Trait-Extract for correction and the “unknown” mixtures to be estimated. This was done for eleven %Trait-Extracts, from 0% to 100% in 10% increments. The PCA method produced an average absolute error of 3.14% (s.d. 1.74%), which was further improved to 1.72% (s.d. 1.37%) by applying the 2^nd^ degree calibration equation nanopore (S6 Table). The maximum deviation was -6.33% before calibration, and -4.27% after calibration.

### Reducing PCR time

To demonstrate that the method is compatible with rapid PCR, reaction conditions were adjusted to complete 35 cycles (i.e., end point) in less than five minutes using assay 14 and a fast PCR device (MBS, PCR Protocol B). During testing of the device, different positions on the 96-well plate were observed to generate different %Trait PCR values. To compensate for this, each PCR pooled the results from three adjacent reaction wells (PCR Protocol B). To test reproducibility, four replicates lanes were run in parallel (S6 Fig). Each replicate lane was used to generate %Trait PCR values for a total of eleven %Trait-Extracts, from 0% to 100% in 10% increments. As before, extracts were made from seed mixtures with the Quick Extraction Protocol.

Using the same protocols used to produce the %Trait PCR values in the reference and test data sets, fast PCR samples can be used to produce test %Trait PCR values. The fast PCR samples were qualitatively analyzed with the Gel Electrophoresis Protocol (S7 Fig), and quantitatively analyzed with the Capillary Electrophoresis Protocol to produce a set of test %Trait PCR values (S7 Table). Reference material was not run on the fast PCR device. To provide a Calibration Equation correction option, the average 50%Trait-Extract PCR value across the 4 replicate lanes (43.9%) was used as a proxy for the 50%Trait-Extract-Mix value, resulting in the 2^nd^ degree equation: y = -0.24664x^2^ + 1.24664x. Similarity of the 50%Trait-Extract and 50%Trait-Extract-Mix value for assay 14 (S2 Table) suggests that the calibration equation should be similar to what would be produced with an averaged 50%Trait-Extract-Mix value generated with the fast PCR device.

When using the 2^nd^ degree equation, the average absolute error between the true %Trait-Extract and the calculated value varied from 2.07%-2.95% across the 4 replicate lanes, with lane 3 showing the largest error of -15.77% at the single value of 60% Trait (suggesting it was an outlier). Combining the calculated %Trait values across the 4 replicate lanes resulted in an average absolute mean error of 1.48% (s.d. 1.71%) and average standard deviation of 2.79% (S7 Table). Thus, with greater redundancy in the workflow, averaging can reduce errors. The largest error was the combined estimate for 60% at -5.32%, again primarily being weighted by the outlier of lane 3. An outlier removal strategy could remedy this issue. As before, we also observe that without calibration the errors are higher: the average absolute error across the 4 replicate lanes is 4.2% (s.d. 3.37%). Across the replicates we also reported bias and coefficient of variation (CV) in S7 Table using a format consistent with [5], but reiterate that scaling by the mean results in relative error comparisons while we focus on the statistics of the absolute error.

## Discussion

The protocol presented here provides a simple method for calculating the relative amount of a transgene, at a unique insertion site, from a weighed sample of seeds. As long as the reference experiments are performed in the same manner as the test experiments, it is also highly accurate across the entire dynamic range (5–100% shown here). All experiments presented were performed manually, with many extraction and PCR mixes formulated independently on different days. It is expected that accuracy would be improved further with automated microfluidics instrumentation, bulk reagents, and a fully characterized and reproducible thermocycling device used for all PCRs.

Although we have used PCR amplified reference samples to generate the calibration equations, this need not be the case once an assay has been defined. We have demonstrated that the same equations can be used to analyze experiments performed at different times and from different seed extractions. We have also shown that as little as one reference data point (namely, the reference 50% Trait reagent) can be used to make a calibration equation that provides accurate results, and additional reference data points were shown to improve accuracy by improving the calibration equation.

While the differences observed when comparing the calculated results with the expected weights are referred to as “errors”, it is worth noting that we cannot be sure that the amplifiable DNA in each individual seed is perfectly correlated with its weight (tests on individual diploid seeds always result in a 0%, 50%, or 100% result). However, on average this appears to be the case when comparing seeds with similar moisture content, as would be expected in containers of seeds.

True errors could also result from a dilution of the extract causing a sampling error. With this in mind, we have tested the dilution limit of the quick extracts, and found that they still give quantitative results when diluted 200,000 times, implying that a 1:100 dilution we use in each of the fast PCR experiments must contain more than 2,000 genome copies. While the maximum sampling error for a sample of 2,000 is +/-2.19% (at a 95% confidence level), each of our measurements was performed on a physical mixture of three independent PCRs, which is expected to bring the potential sampling error down closer to +/-1%.

Designing 3-primer assays for quantification is as simple as designing standard 2-primer PCR assays, and a large majority of primer sets that we have tested have produced working assays with little optimization. Indeed, the three 3-primer assays presented here for the GTS40-3-2 event all worked with a common PCR protocol. By contrast, methods that use isothermal amplification require more complicated primer sets that loop or contain restriction sites, and optimization is not as straightforward.

Here we have used two different technologies to measure the ratio of the two PCR amplicons, capillary electrophoresis and solid-state nanopores, with comparable results. In practice, a number of other methods could be used to measure the ratio, such as gel electrophoresis, sequence specific fluorescent probes, or separation of the molecules with affinity tags on the primers, followed by quantification. For technologies where the separation of molecules must be compared to a reference ladder, the optimal 50% reference sample can be synthetically made from the proper amounts of the two DNA molecules, and can be run before or after the test sample.

Where bulk sample testing in the field is desired, the compatibility of the method with non-optical solid-state nanopore sensors is attractive. By combining quick extraction and fast PCR with nanopore measurement and data analysis, one can achieve accurate results fast. To that end, we also developed a single-control based quantification that uses principal component analysis applied to the nanopore data (S2 Text). Only a single control mixture is required (nominally, the 50%Trait), which can be recorded during sub-5 min PCR of the test sample, followed by nanopore measurement of the PCR product, for a seed-to-answer result in less than 10 minutes. As all of the reagent components including the single polymerase enzyme are commercially available in bulk, are thermostable, can be lyophilized, and are not sensitive to light, production of assays would be uncomplicated, and storage and shipping of assays could be inexpensive at appreciable volumes.

As long as the protocol is consistent between the reference samples and the test samples, the quick extraction protocol presented can also be modified. For example, we have tested a two-step extraction, where the 35g of seeds are first extracted using only water, and a small amount of that was then mixed with a NaOH or detergent containing solution, and then that small volume was diluted and/or neutralized before use a PCR template. These variations were fully compatible with the method, and reduced the amount of chemicals necessary, thus lowering the cost and environmental impact.

While we have presented only a single use case, the method is very general and is not limited to quantitative analysis of transgenes in mixtures of seed crops. Using the same PCR method, determination of zygosity of a transgene (or any chromosome rearrangement, natural or introduced) in individual organisms would be straightforward. Unlike traditional zygosity assays, which typically give a Y/N readout, the presented method could also be used to determine zygosity in polyploid organisms. It could also be used to quantify the frequency of a deletion, insertion, or rearrangement in a population of haploid organisms or organelles, such as bacteria, mitochondria, and chloroplasts.

## Supporting information

S1 Fig Qualitative gels of sixteen assays tested with 0%Trait-Extract, 50%Trait-Extract, and 100%Trait-Extract. (TIF)

S2 Fig Specificity test of assays 2, 14 and 16 with templates made from 0%Trait, 50%Trait, and 100%Trait seed mixes. (TIF)

S3 Fig Qualitative gel of Experiments A (left column) and B (right column) for Assays 2, 14 and 16.
(TIF)

S4 Fig Qualitative gel of assay 2 Experiment C. (TIF)

S5 Fig Qualitative gel of assay 2 Experiments C1-C3 (for comparison purposes, C1 is the same as Experiment C in S4 Fig). (TIF)

S6 Fig Replicate lanes of triplicate PCR wells for fast PCR workflow. (TIF)

S7 Fig Qualitative gel of assay 14 fast PCR samples (MBS device, PCR Protocol B) for Replicate Lanes 1–4 (S5 Fig) products aligned vertically, at each of the %Trait-Mix values assayed. (TIF)

S1 Table Sixteen different three-primer assays for the model Trait vs. Non-Trait system demonstrated. (XLSX)

S2 Tables Capillary electrophoresis protocol applied to 50% Trait mixtures for the sixteen different three-primer assays following end-point PCR. (PDF)

S3 Tables Data and calculated Calibration Equations for assays 2, 14 and 16 using Experiment A and B data permutations. (XLSX)

S4 Table The DNA sequences. (PDF)

S5 Table Triplicate repeats of experiment C data and analysis results. (XLSX)

S6 Table Nanopore-based quantification results for of experiment C data, and nanopore sizes. (XLSX)

S7 Table Quantitative results of applying the capillary electrophoresis protocol to produce %Trait PCR values for the fast PCR products. (XLSX)

S1 Raw Images Single PDF file that contains all the original blot and gel images contained in the manuscript’s main figures and supplemental figures. (PDF)

S1 Text Solid-state nanopore chip fabrication and injection molded test strip creation and testing methods. (PDF)

S2 Text Trait vs. non-trait relative quantification from nanopore data methods. (PDF)
