# Correction: Fast and accurate quantification of insertion-site specific transgene levels from raw seed samples using solid-state nanopore technology

**DOI:** 10.1371/journal.pone.0229524

**Published:** 2020-02-18

**Authors:** The PLOS ONE Staff

The second author’s name is spelled incorrectly. The correct name is: Leslee T. Nguyen. The correct citation is: Pearson MD, Nguyen LT, Zhao Y, McKenna WL, Morin TJ, Dunbar WB (2019) Fast and accurate quantification of insertion-site specific transgene levels from raw seed samples using solid-state nanopore technology. PLoS ONE 14(12): e0226719. https://doi.org/10.1371/journal.pone.0226719. The publisher apologizes for the error.
