# Supplementary figures and images for: Fast and accurate quantification of insertion-site specific transgene levels from raw seed samples using solid-state nanopore technology
Source: PLoS One. 2019 Dec 27;14(12):e0226719. doi: 10.1371/journal.pone.0226719 (PMC6934305; doi:10.1371/journal.pone.0226719)

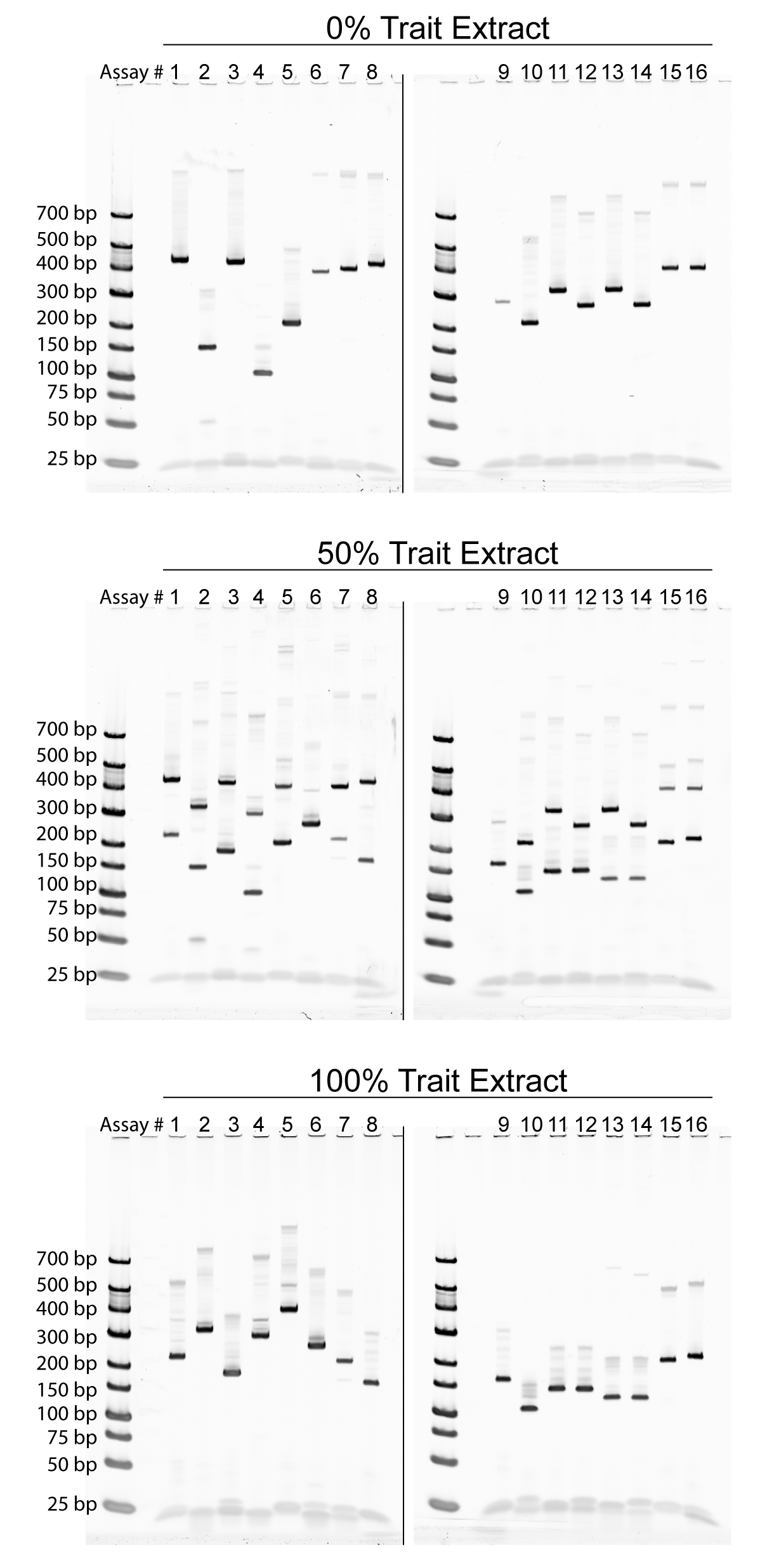

Supplement: S1 Fig — (TIF) [file pone.0226719.s001.tif]

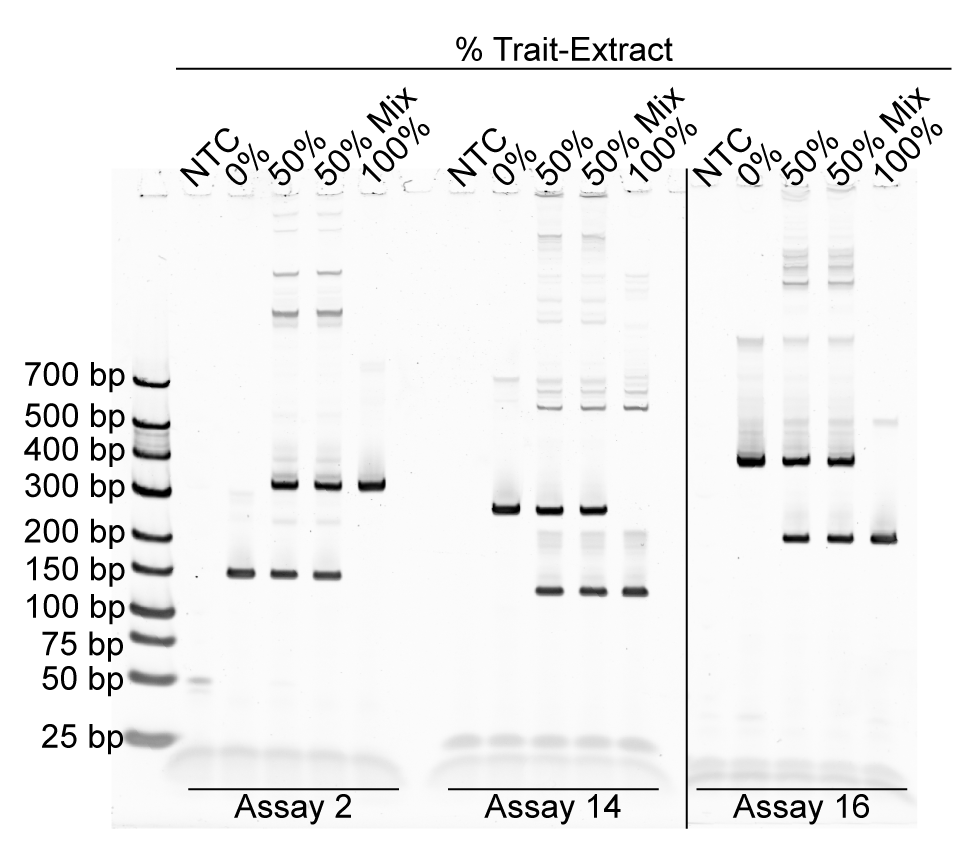

Supplement: S2 Fig — (TIF) [file pone.0226719.s002.tif]

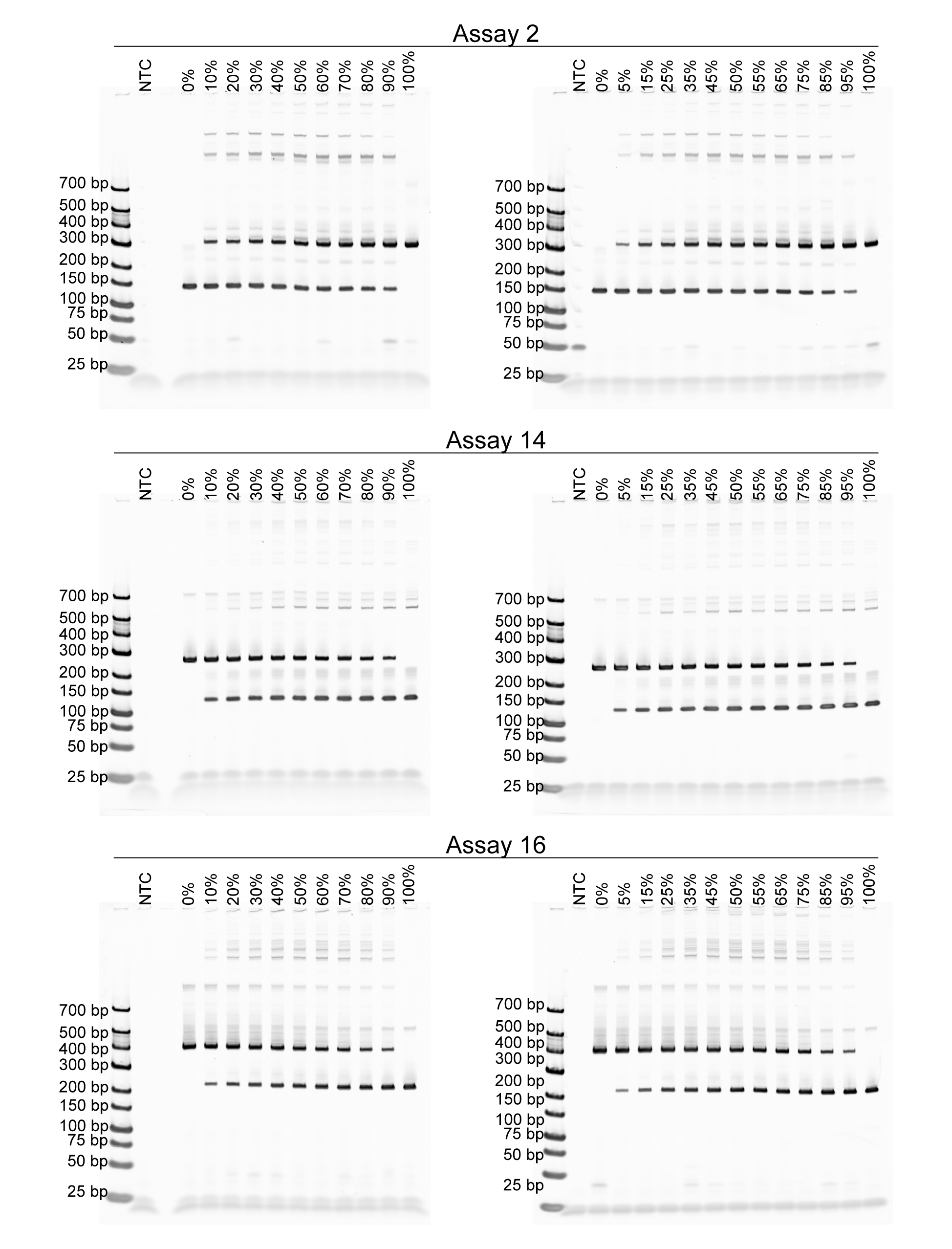

Supplement: S3 Fig — Qualitative gel of Experiments A (left column) and B (right column) for Assays 2, 14 and 16. (TIF) [file pone.0226719.s003.tif]

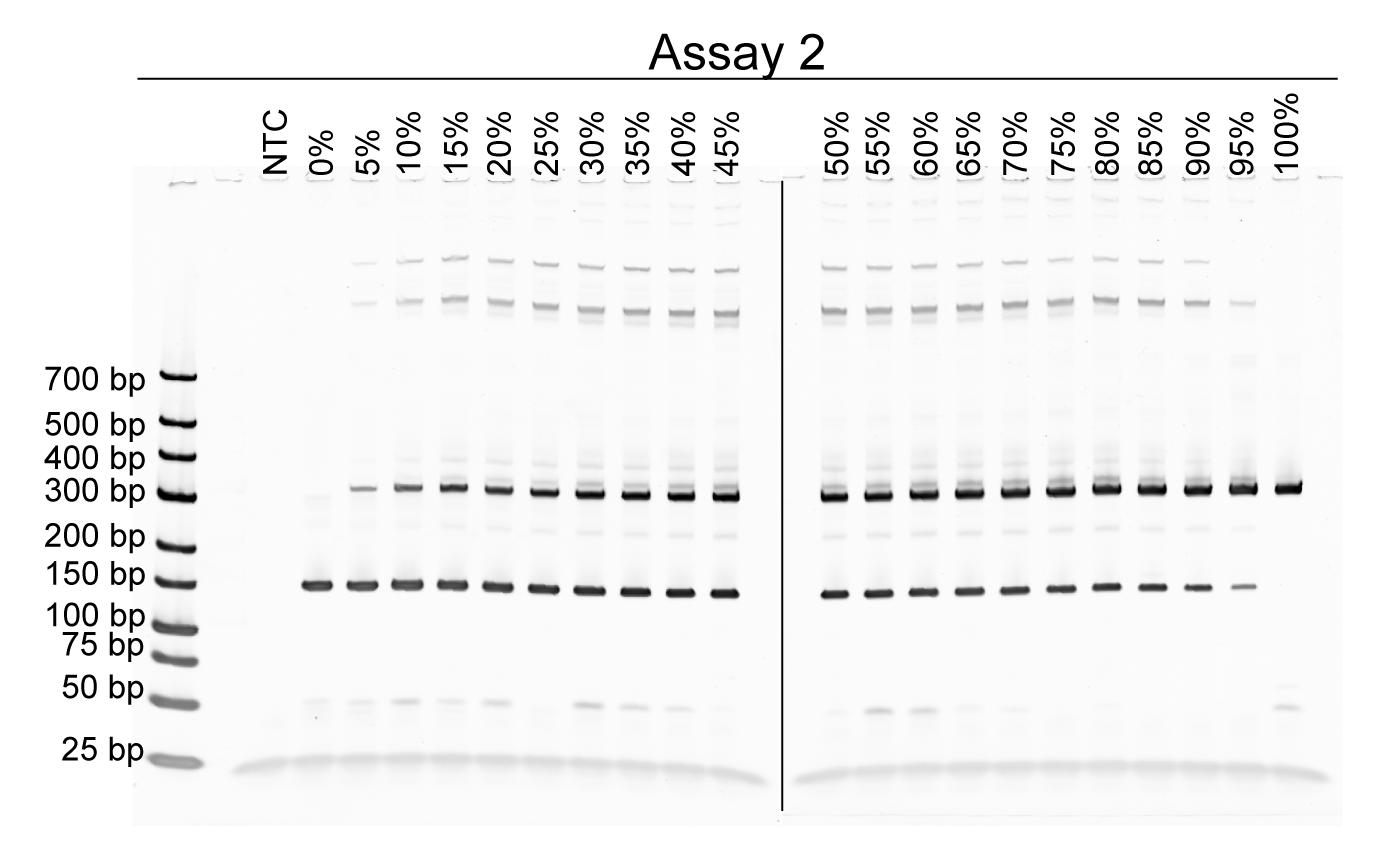

Supplement: S4 Fig — (TIF) [file pone.0226719.s004.tif]

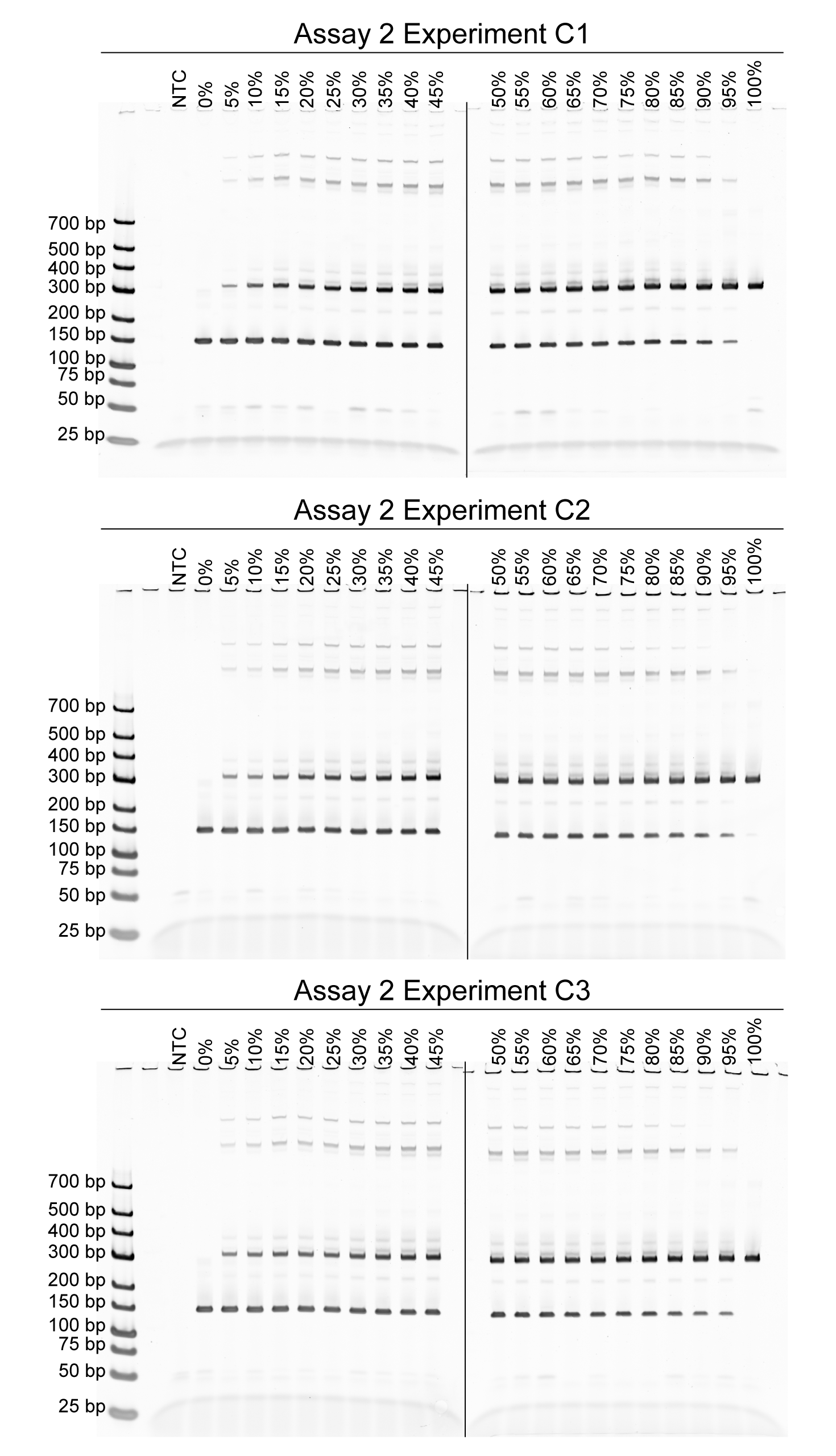

Supplement: S5 Fig — (TIF) [file pone.0226719.s005.tif]

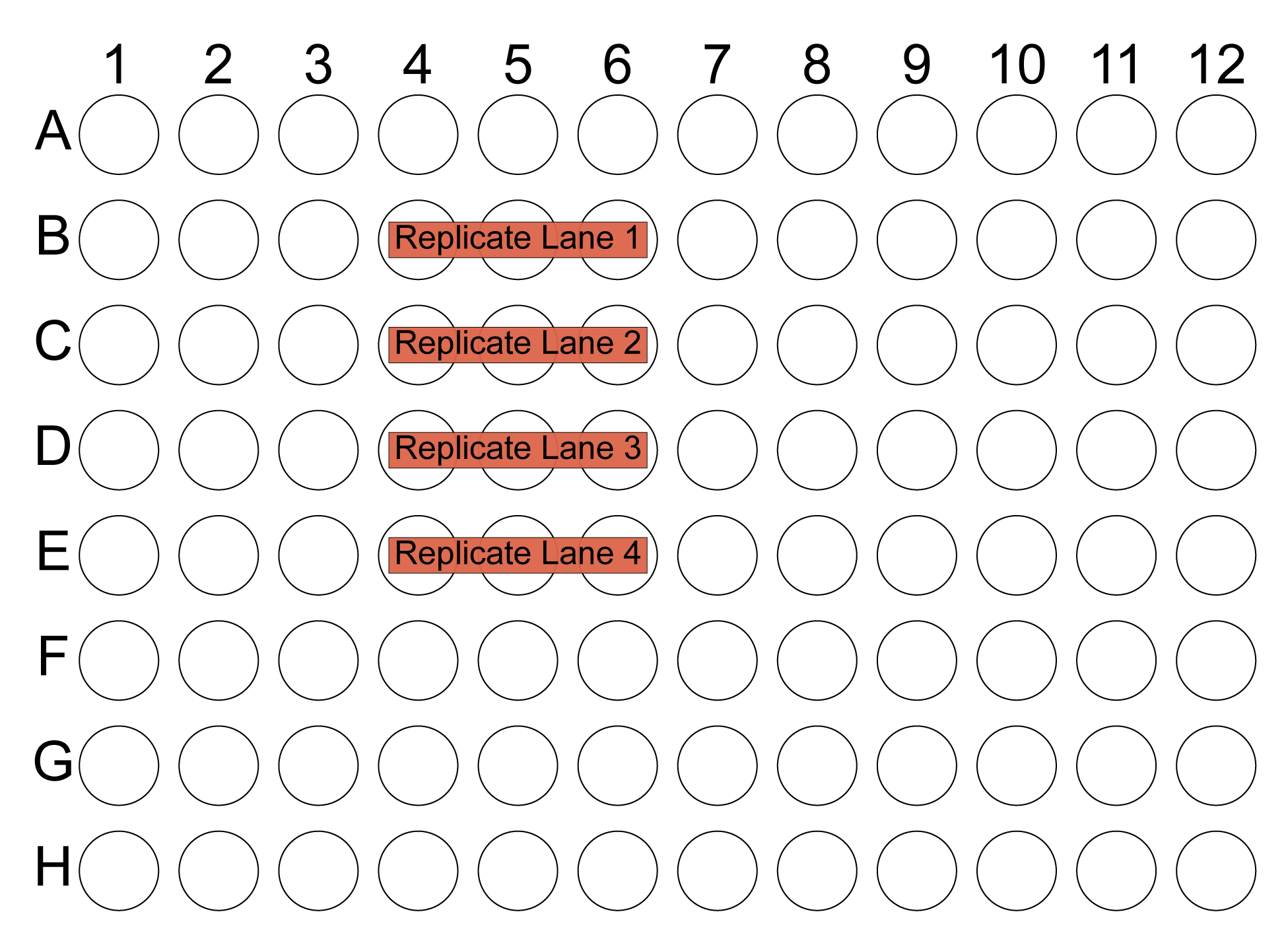

Supplement: S6 Fig — (TIF) [file pone.0226719.s006.tif]

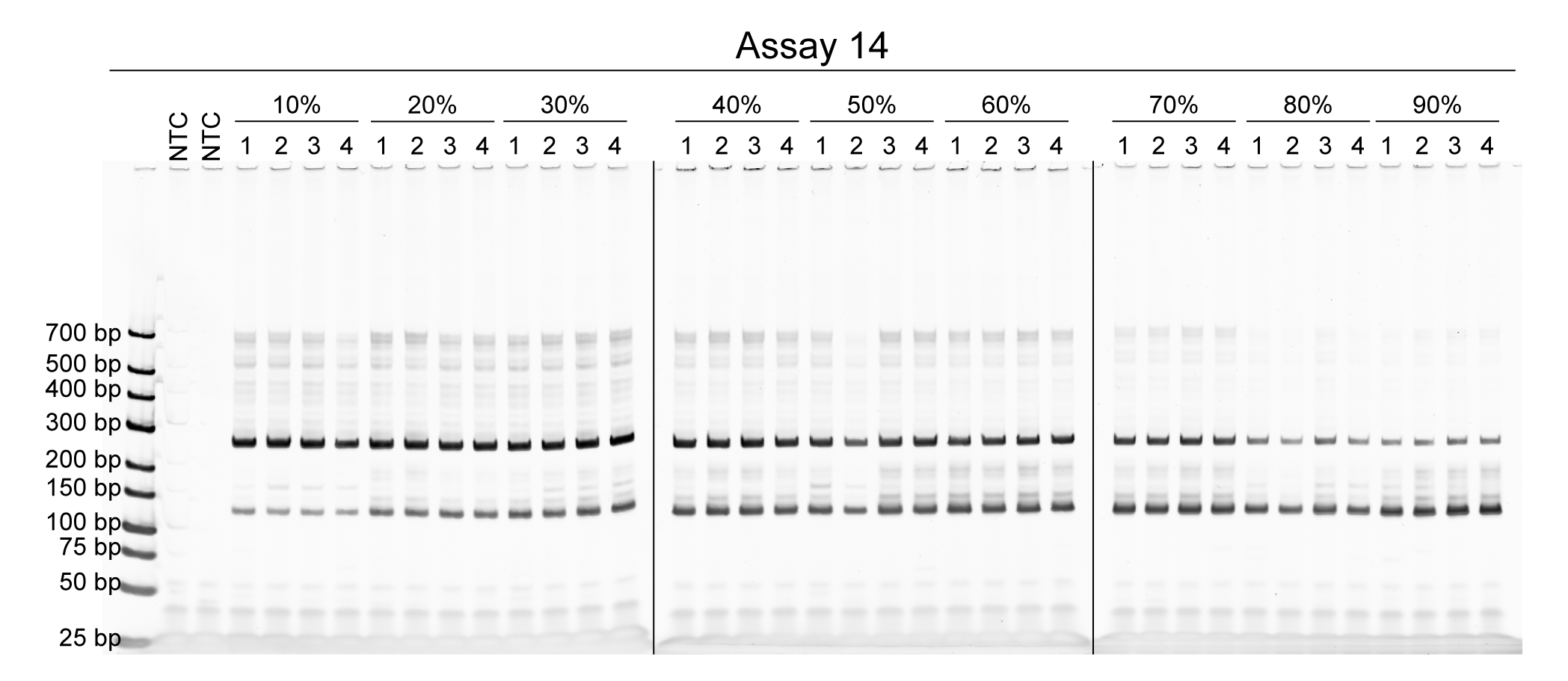

Supplement: S7 Fig — (TIF) [file pone.0226719.s007.tif]
