# Supplementary material for: Fast and accurate quantification of insertion-site specific transgene levels from raw seed samples using solid-state nanopore technology
Source: PLoS One. 2019 Dec 27;14(12):e0226719. doi: 10.1371/journal.pone.0226719 (PMC6934305; doi:10.1371/journal.pone.0226719)
Supplement: S2 Tables — (PDF) [file pone.0226719.s009.pdf]

Tables S2

a

	%Trait-PCR		
	**Assay 2**	**Assay 14**	**Assay 16**
**0%Trait-Extract**	0.00%	0.00%	0.00%
**50%Trait-Extract**	51.00%	38.10%	46.60%
**50%Trait-Extract-Mix**	47.80%	42.30%	48.70%
**100%Trait-Extract**	100.00%	100.00%	100.00%

b

Assay	50% Trait-Extract
**1**	30.0%
**2**	55.8%
**3**	41.2%
**4**	60.5%
**5**	40.4%
**7**	29.4%
**8**	29.4%
**10**	37.0%
**11**	50.0%
**12**	58.5%
**13**	37.1%
**14**	43.8%
**15**	60.8%
**16**	66.4%
