# Supplementary material for: Fast and accurate quantification of insertion-site specific transgene levels from raw seed samples using solid-state nanopore technology
Source: PLoS One. 2019 Dec 27;14(12):e0226719. doi: 10.1371/journal.pone.0226719 (PMC6934305; doi:10.1371/journal.pone.0226719)
Supplement: S4 Table — (PDF) [file pone.0226719.s011.pdf]

```
>GTS 40-3-2 RoundUp-Ready Junction A
AATTAAATAAATCAATTACTTCATAAATAATTTTTTTTATAGAATATGTT
GACATTCTAGCCGGATATAGAACTAATGTAAAGAAACCTTAAAAATTTTG
TTTGGAAGAATATGTTATTGAAAGACAAATCTAATTAAGTTTATCAGGGT
CATTTGTTGAAGATAGGAAACCTTCAGCAATTTGAATATTAAGTAACTGC
TTCTCCCAGAATGATCGGAGTTTCTCCTCCTGCTATTACATGAGCAAAAA
TAAAAAATAAATAAAAGATAAGATTAAGCTTCAACATGTGAAGGAGTAGT
ACACTCACCAGTGACCCTAATAGGCAACAGCATGAAAAAAAATAAAAAAG
AATAAAAATAGCATCTACATATAGCTTCTCGTTGTTAGAAAAACAAAACT
ATTTGGGATCGGAGAAGAACTGTTTGAGGCGAATGGCCTGGTCGTCGCGG
CCATCGTCGAGAAGTTCGTGAAGAAGCTCGAATGCGGTGAGAAGGTAGTT
CTCTTCCAACAGAAAGTTCACCACGCAATTGCACAGCGAAGATCTCTCCA
CGTCCATTTTCTCTCTCTGTCTCTGATCTTAAGCCATTCATTCAAGACAA
GACAAGAGAAGAGAAGAGAAGAGAAGAGAACACTCTCAGTCAGATCGTGG
TTTCAACTTTCAAGACTGTGCTAGCTAGTTAGGTGCCATCTTACATGTTT
ACTTTTTTTCTTTATAAGATTAAATTGCTGAATACCATGCTCTCCTGTGT
CCAAAGCAGTACACCCGCGTAAAAATAGATTTCATCGTCCTTTCGATTTT
AC
>GTS 40-3-2 RoundUp-Ready Junction B
GAAACCGCGTAATTTTGGAGCTATGCTTCAGTAAATTTAAAATATTATCC
TTCAGATACAATATATATATATATATATATATATATATATATATATATTA
TCCTTCAATCTGCTTAACATATTTTTATCTGAATCCCTACATTGCGATTC
TCGATCTTACCGAATCCTTATTATTTATGTTTAAATTTTGCCCCTCTTTT
TTTCATGCTTTATTTTTTACAATGAACAAGATTTGAATTCAGAACCTTGT
GCAAATTATTCAAACCCTTCAATTTAACCGATGCTAATGAGTTATTTTTG
CATGCTTTAATTTGTTTCTATCAAATGTTTATTTTTTTTTACTAGAAATA
ACTTATTGCATTTCATTCAAAATAAGATCATACATACAGGTTAAAATAAA
CATAGGGAACCCAAATGGAAAAGGAAGGTGGCTCCTACAAATGCCATCAT
TGCGATAAAGGAAAGGCCATCGTTGAAGATGCCTCTGCCGACAGTGGTCC
CAAAGATGGACCCCCACCCACGAGGAGCATCGTGGAAAAAGAAGACGTTC
CAACCACGTCTTCAAAGCAAGTGGATTGATGTGATATCTCCACTGACGTA
AGGGATGACGCACAATCCCACTATCCTTCGCAAGACCCTTCCTCTATATA
AGGAAGTTCATTTCATTTGGAGAGGACACGCTGACAAGCTGACTCTAGCA
GATCTTTCAAGAATGGCACAAATTAACAACATGGCACAAGGGATACAAAC
CCTTAATCCCAATTCCAATTTCCATAAACCCCAAGTTCCTAAATCTTCAA
>Insertion-site A chr2:7841570..7842369
AAGTCCCCATAGATTACATAACCGACAAAAACAATGCCCATATCTAGGAA
GCCAATACAGTCGATATAAATAACATTAATCCACACCTAAATGTCATAAC
TCATAAACAACCCTAAG[C/T]ATTAAATTGGAGTCCAAGTACTAGAGAA
AGGCTTAATTTCGTATTGTAATCTCCCTCAGAATTTCTTAATCTTGTGAT
CAACAAAGCATATC[C/G]TCGTTTT[A/C]AATTCTAAAGGTTATGGCA
AAATTCACTGGCATACGAACAATTCATATATCCATTCCTATTATATATAG
TTGGCAGAAGTACAAGGAGGCGCCAAATAGAAAACACAAATTGGAACGGT
GAAGAGAAAGAAGAGTACCTCGGAGAGAGTTGAGGCGAGAGATGAGATCG
GGAGGGAAGAGATTGGGATCGGAGAAGAACTGTTTGAGGCGAATGGCCTG
GTCGTCGCGGCCATCGTCGAGAAGTTCGTGAAGAAGCTCGAATGCGGTGA
GAAGGTAGTTCTCTTCCAACAGAAAGTTCACCACGCAATTGCACAGCGAA
GATCTCTCCACGTCCATTTTCTCTC[T/C]CTGTCTCTGATCTTAAGCCA
TTCATTCAAGA[C/G]AAGA[C/G]AAGAGAAGA[G/C]AAGAGAA[G/C]
AGAAGAGAACACTCTCAGTCAGATCGTGGTTTCAACTTTCAAGACTGTGC
TAGCTAGTTAGGTGCCATCTTACATGTTTACTTTTTTTCTTTATAAGATT
AAATTGCTGAATACCATGCTCTCCTGTGTCCAAAGCAGTACACCCGCGTA
AAAATAGATTTCATCGTCCTTTCGATTTTAC
>Insertion-site B chr2:8002760..8001961
G[A/G]AACCGCGTAATTTTGGAGCTATGCTTCA[G/A]TAAATTTAAAA
TATTATCCTTCAGATACAATATATATATATATATATATATATATATATAT
ATATATTATCCTTCAATCTGCTTAACATATTTTTATCT[G/A]AATCCCT
ACATTGCGATTCTCG[A/G]TCTTA[C/T]CGAATCCTTATTATTTATGT
TTAAATTTTGCCCCTCTTTTTTTCATGCTTTATTTTTTACAATGAACAAG
ATTTGAATTCAGAACCTTGTGCAAATTATTCAAACCCTTCAATTTAACCG
ATGCTAATGAGTTATTTTTGCATGCTTTAATTTGTTT[C/T]TATCAAAT
GTTT[A/T][T/A]TTTTTTTTACTAGAAATAACTTATTGCATTTCATTC
AAAATAAGATCATACATACA[G/T]GTTAAAA[T/A][A/T]AAAAAA
[A/T/G/C][T/G]GCAGTAATTAGCATAGAAACGAGGTGCTCTTGCAAA
GATGTGAACGACACGATTGACTTGCCGCTTGATAAAACTAACCTTATAGA
TCAAATGTTTTAAAAACTAATAATCCAGAACTTAATACTATGAAC[A/C]
TTATAAATCATATTTAAACTTTTTT[T/G]AATAAAGGAATTTGTTAATG
TATAATGATTTAGGACATGTTTGGTTAACT[G/A]TTCAAAAAGT[A/C]
CTTTTGAATGCTAAACATAGTTTGGTTATCCTCAAAACTATGTGCAACTA
AAATTTTGTCTGAAAAC[T/A]ACTCAATTTCGTAGTACACTAAGTTATT
TTTGCAAACTCAATTTACTAACTTAAATTTAACTCAAACTTAAATTTACA
AGCTTTAATCTAAACATGCATTTAGTGT
```
