# Supplementary material for: Fast and accurate quantification of insertion-site specific transgene levels from raw seed samples using solid-state nanopore technology
Source: PLoS One. 2019 Dec 27;14(12):e0226719. doi: 10.1371/journal.pone.0226719 (PMC6934305; doi:10.1371/journal.pone.0226719)
Supplement: S1 Raw Images — (PDF) [file pone.0226719.s015.pdf]

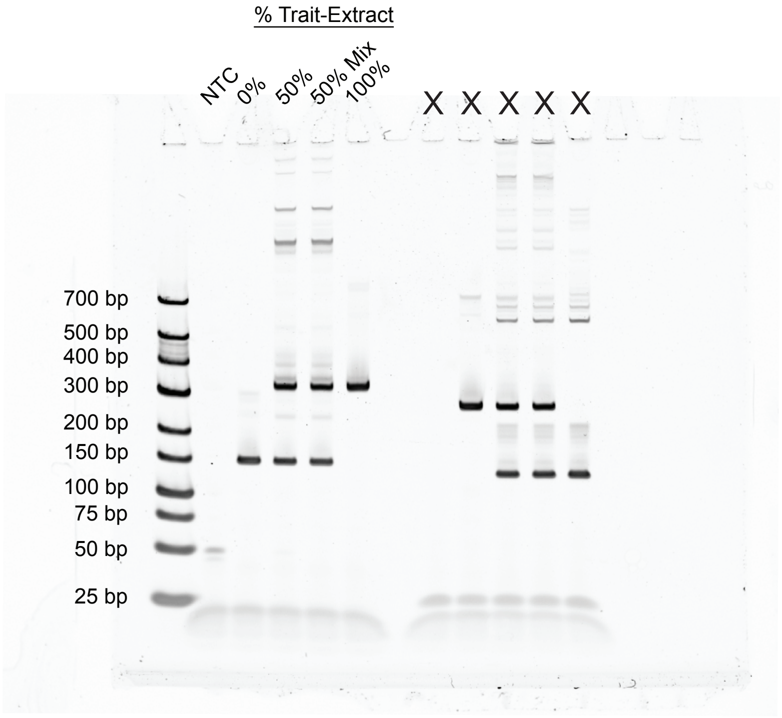

Figure 1. Specificity of assay 2

6% TBE PAGE gel run at 200V for 25 minutes

Stained with SYBR Green for 15 minutes

Imaged using Bio-Rad ChemiDoc MP

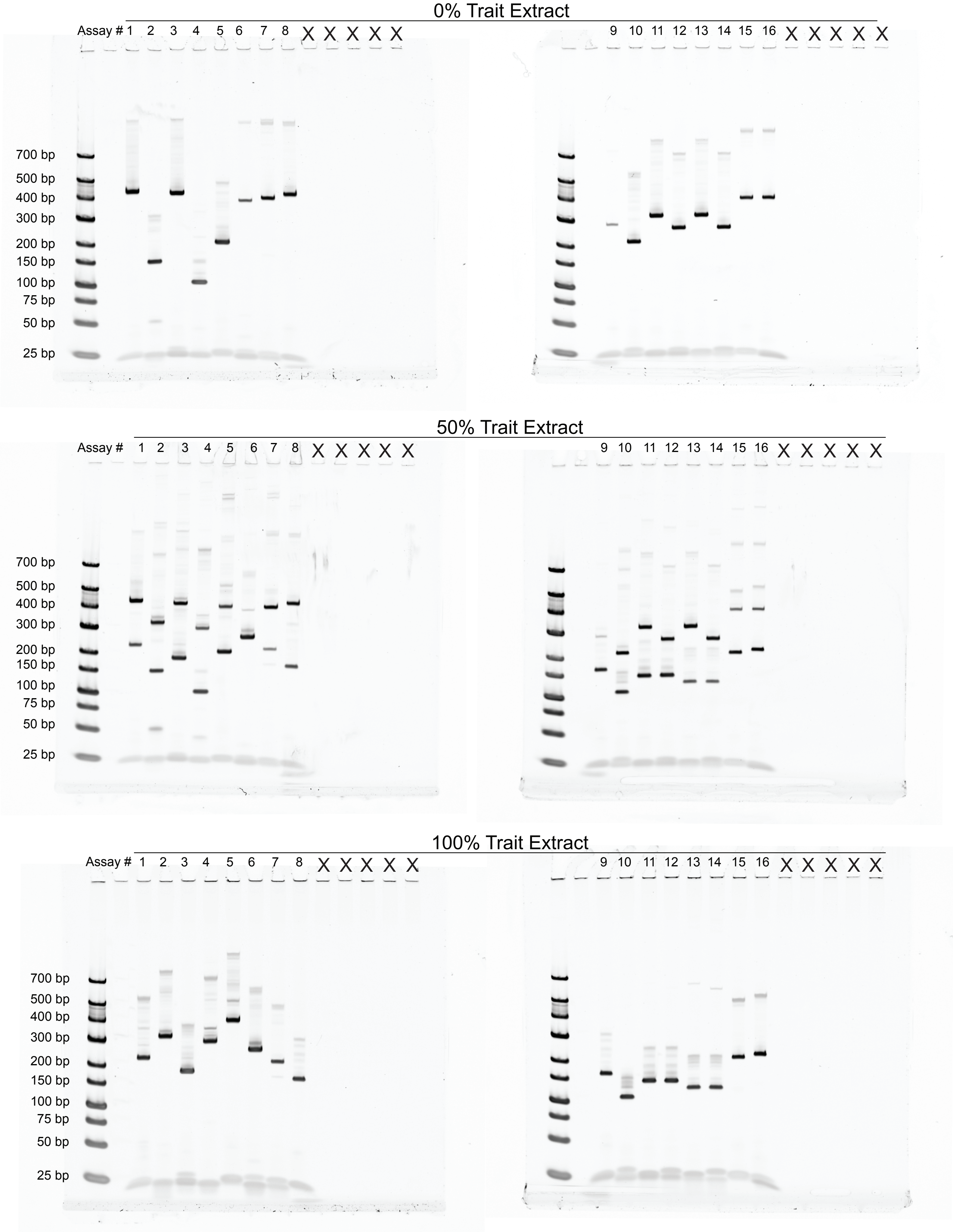

<u>Figure S1. Qualitative gels of sixteen assays tested with 0%, 50%, and 100% Trait-Extract</u>
6% TBE PAGE gel run at 200V for 25 minutes
Stained with SYBR Green for 15 minutes
Imaged using Bio-Rad ChemiDoc MP

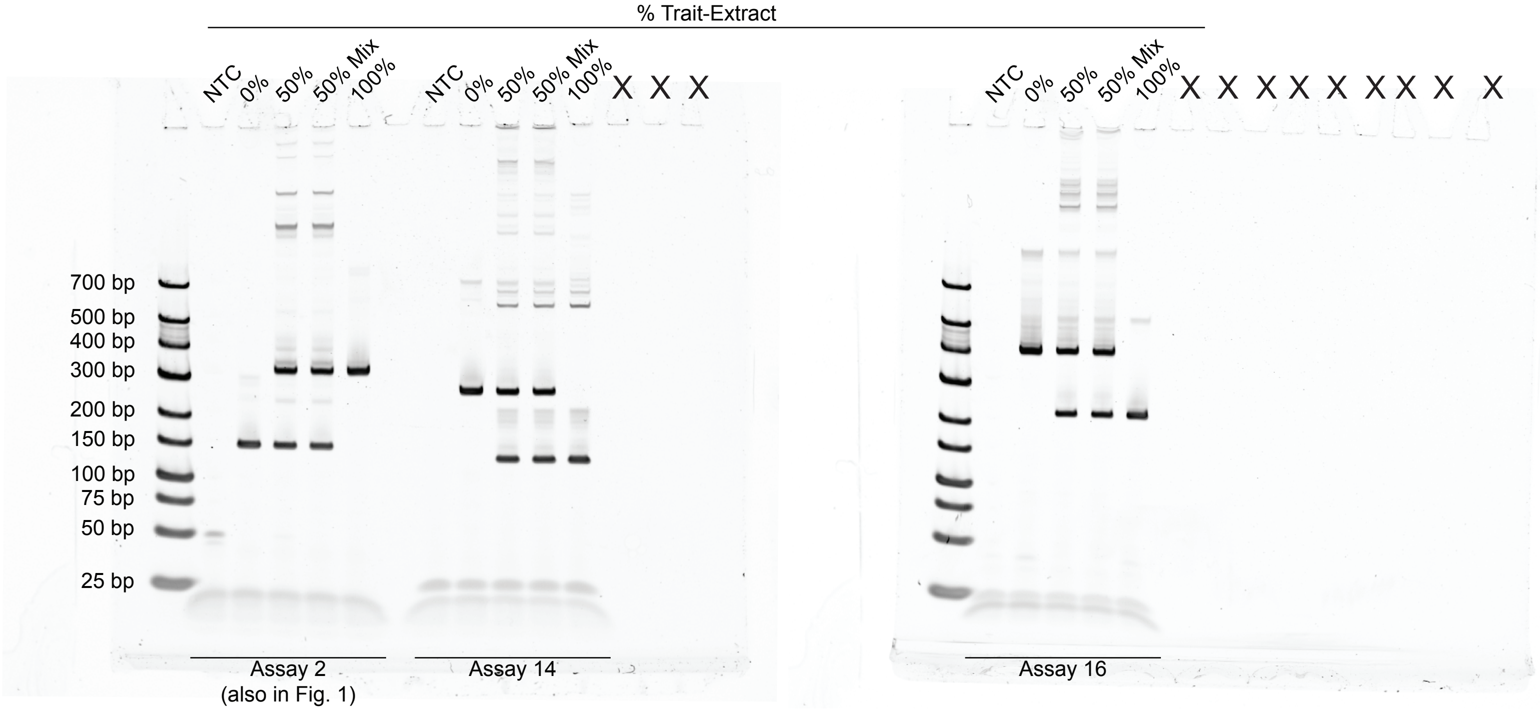

<u>Figure S2. Specificity test of assays 2, 14, and 16 with templates made from 0%, 50%, and 100% Trait Extract and Extract-Mixes</u>

6% TBE PAGE gel run at 200V for 25 minutes
Stained with SYBR Green for 15 minutes
Imaged using Bio-Rad ChemiDoc MP

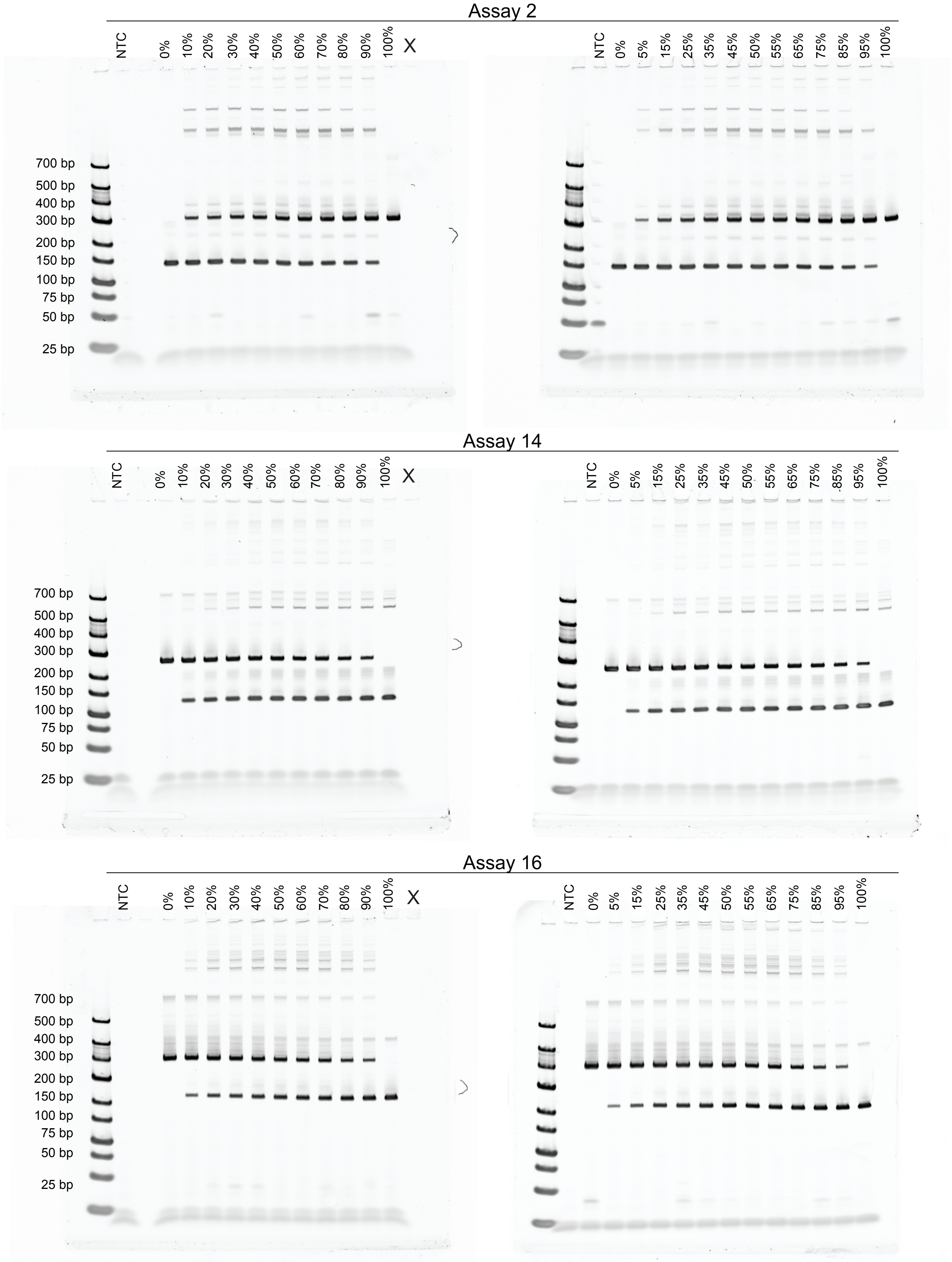

Figure S3. Qualitative gels of Experiments A (left) and B (right) for assays 12, 14, and 16
6% TBE PAGE gel run at 200V for 25 minutes
Stained with SYBR Green for 15 minutes
Imaged using Bio-Rad ChemiDoc MP

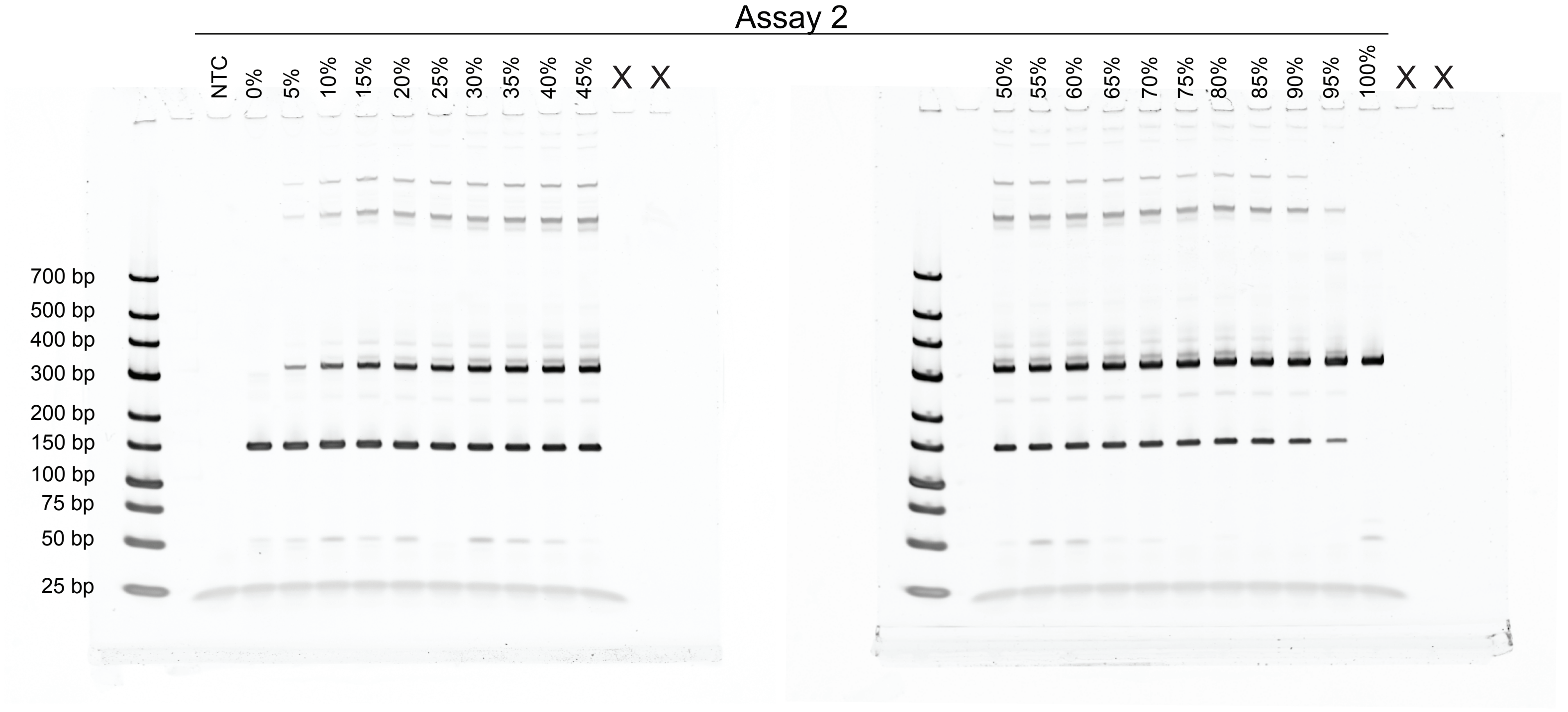

<u>Figure S4. Qualitative gel of assay 2 experiment C</u>
6% TBE PAGE gel run at 200V for 25 minutes
Stained with SYBR Green for 15 minutes
Imaged using Bio-Rad ChemiDoc MP

Assay 2 Experiment C1 (also in Fig. S4)

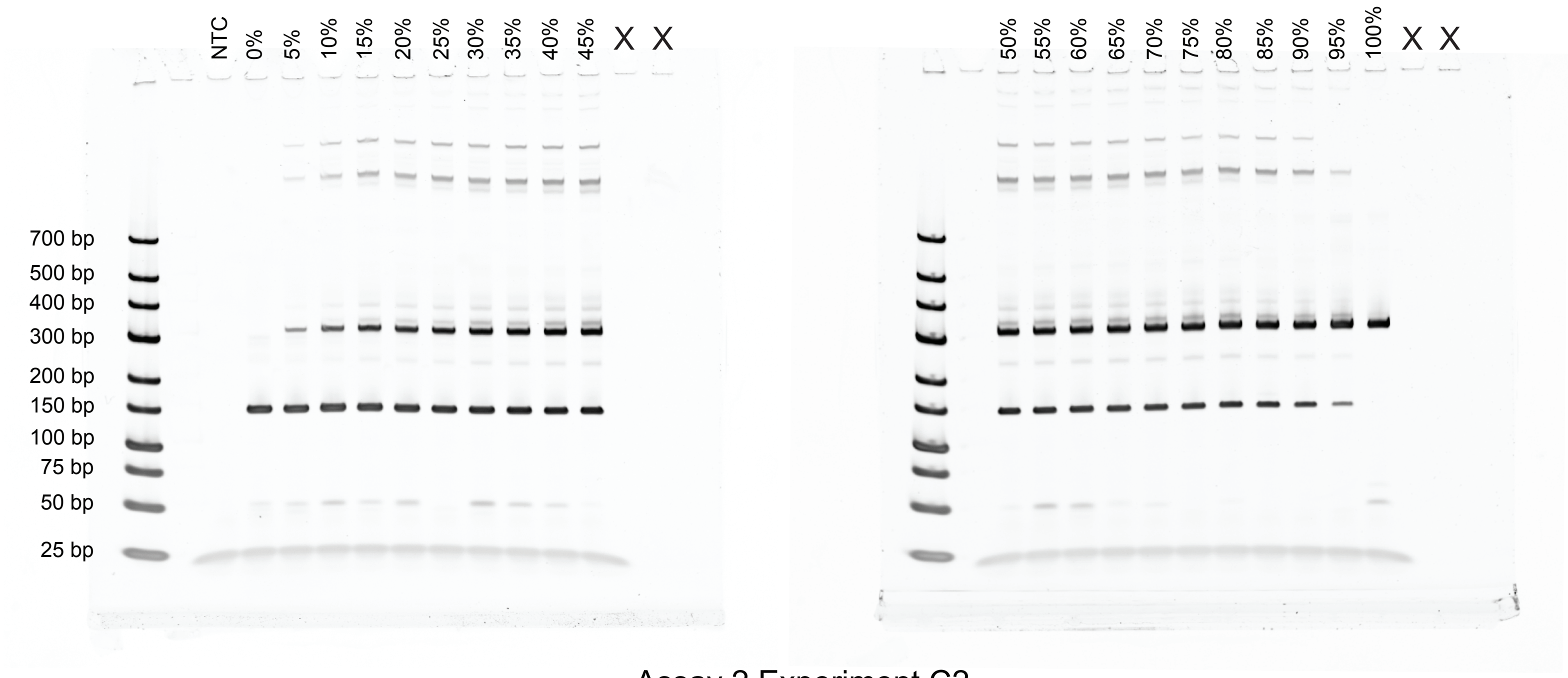

Assay 2 Experiment C2

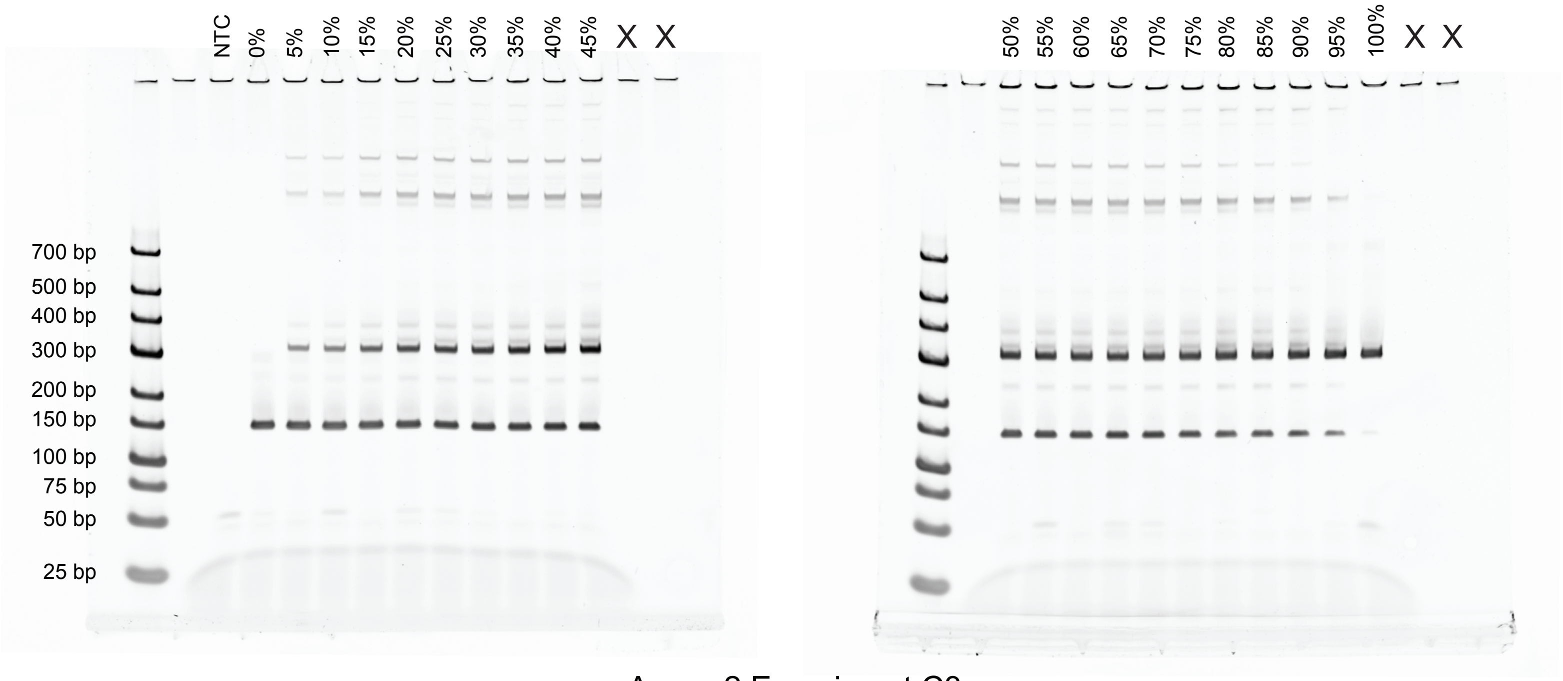

Assay 2 Experiment C3

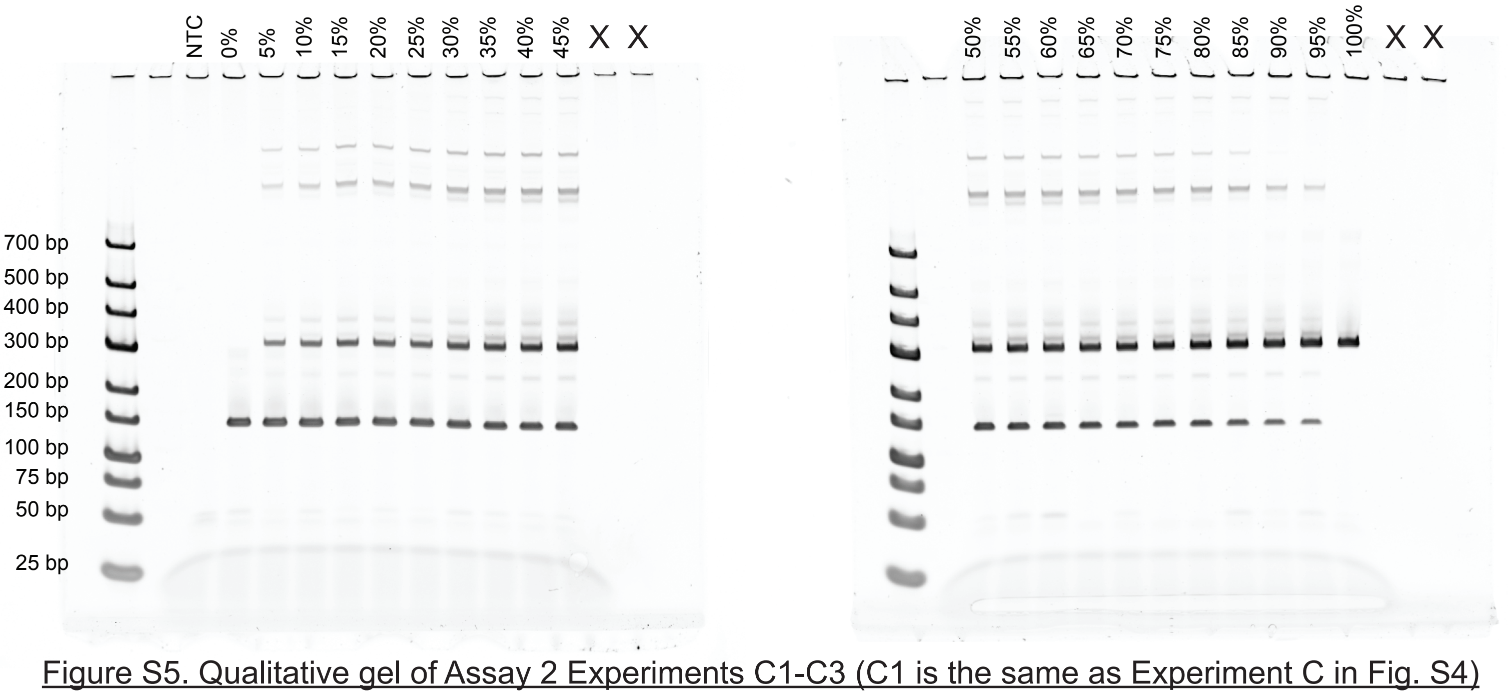

Figure S5. Qualitative gel of Assay 2 Experiments C1-C3 (C1 is the same as Experiment C in Fig. S4)

6% TBE PAGE gel run at 200V for 25 minutes

Stained with SYBR Green for 15 minutes

Imaged using Bio-Rad ChemiDoc MP

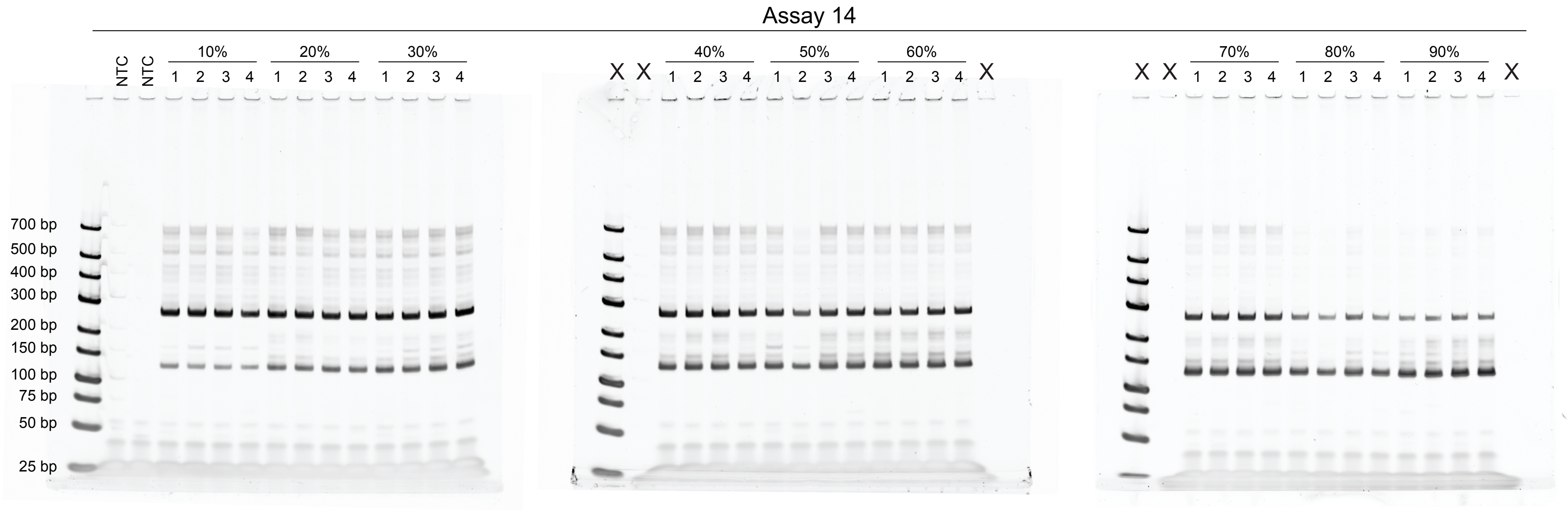

Figure S7. Qualitative gel of Assay 14 fast PCR samples (MBS device, PCR protocol B)
6% TBE PAGE gel run at 200V for 25 minutes
Stained with SYBR Green for 15 minutes
Imaged using Bio-Rad ChemiDoc MP
