# Supplementary material for: Fast and accurate quantification of insertion-site specific transgene levels from raw seed samples using solid-state nanopore technology
Source: PLoS One. 2019 Dec 27;14(12):e0226719. doi: 10.1371/journal.pone.0226719 (PMC6934305; doi:10.1371/journal.pone.0226719)
Supplement: S1 Text — (PDF) [file pone.0226719.s016.pdf]

Supporting Information for

"Fast and Accurate Quantification of Insertion-site Specific Transgene Levels from Raw Seed Samples using Solid-State Nanopore Technology"

The nanopore chip

First, 30 nm of low-stress low-pressure CVD (LPCVD) SiN thin film (<200 MPa, tensile) is deposited on a 750 um Si substrates (Thermco LPCVD Nitride). The nanopores are formed in the SiN membrane by first patterning with PMMA and then exposing the 30 nm nanopore pattern using electron beam lithography (EBL) (JEOL JBX-6300 Lithography System) followed by reactive ion etching of the nanopore (RIE Oxford PlasmaPro 80). After the etch the final diameter falls within 25-35 nm. To reduce noise, an insulating layer, consisting of 1 um SiO2 layer, was deposited on the front side of the wafer using a plasma-enhanced CVD (PECVD) (PlasmaTherm Shuttlecock PECVD System) process followed by a 1000 C anneal for one hour (Thermco Oxidation Furnace). An additional 400 nm SiN etch mask layer was deposited via LPCVD (Thermco LPCVD Nitride) on the substrate following the anneal. The etch pit was opened from the backside by photolithography followed by reactive ion etching of the SiN etch mask layer (RIE Oxford PlasmaPro 80). A second photolithography step was performed on the front side of the wafer to define the SiO2 micro-well pattern. Subsequently, reactive ion etching (RIE Oxford PlasmaPro 80) was used to partially open the SiN mask and SiO2 layer with target etch depth of 0.8 um. The SiN membrane was then fully released by removing the remaining oxide and Si material from both sides of the wafer using a KOH wet etch. First, while protecting the frontside of the wafer, a KOH wet etch removed the Si substrate from the etch-pit side. Second, while protecting the backside, another KOH wet etch removed the the remaining oxide material from the frontside, fully releasing the SiN membrane and nanopore. A schematic of the nanopore chip is shown in Fig 1.

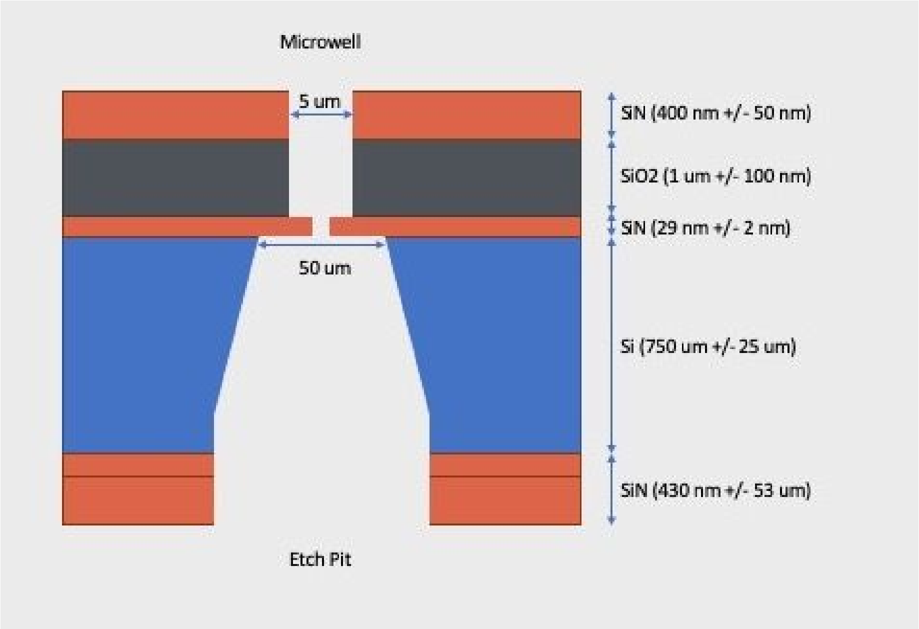

Fig 1. Schematic of fabricated solid-state nanopore chip. The nanopore diameter is within 25-35nm across an entire wafer.

Injection molded test strip - assembly and use

The test strip is shown schematically in exploded and assembled views in Fig 2. The test strip top and base are injection molded in clear Polycarbonate (Makrolon 2407-5500115). The test strip chip and channel seal is injection molded in elastomer (211-45 Santoprene). The electrodes are screen printed Ag/AgCl ink (Creative Materials 113-09S) on 5 mil PET sheeting with an anti-abrasive Carbon coating on the connecting end (Creative Materials 124-50T). Prior to assembly, polycarbonate and elastomer parts are cleaned with 99.5% IPA in an ultrasonic cleaner (Digital Pro+) for three (3) minutes, flushed with deionized (DI) water, and dried in a food dehydrator (Excalibur 2900ECB) at ambient temperature. The nanopore chip and channels are sealed by compression of the central elastomer seal. Two screws and nuts are tightened to 6 ozf.in. with a calibrated torque screwdriver (Mountz TLF-IFR) for even compression of the seal to 25% to ensure leak-free sealing such that electrical conductivity between the channels can occur only through the nanopore. Each channel is approximately 8 microliters in volume.

Prior to reagent testing, assembled test strips were prepared as follows. Test strips were filled with 10 uL of buffer in both the cis and trans channels, and the strips were loaded into the custom voltage-clamped amplifier [1] . Square voltage pulses 0.2 s in duration and ranging from ±2V to ±12V in magnitude were used to incentivize nanopore wetting. Following wetting, nanopore fitness was assessed by the symmetry of conductance over a voltage sweep from -0.3V to 0.3V, and by the root-mean-square of the current (IRMS) at 0.1 V. Pores with asymmetry < 10% and IRMS < 30 pA were used for reagent testing. Nanopore sizes estimated from the current, following the method detailed in [2], ranged from 25-35 nm at the start of

reagent testing. Nanopores grew up to 40 nm in diameter in some cases during the process of reagent testing, for a total diameter range of 25-40 nm across all data provided in the paper.

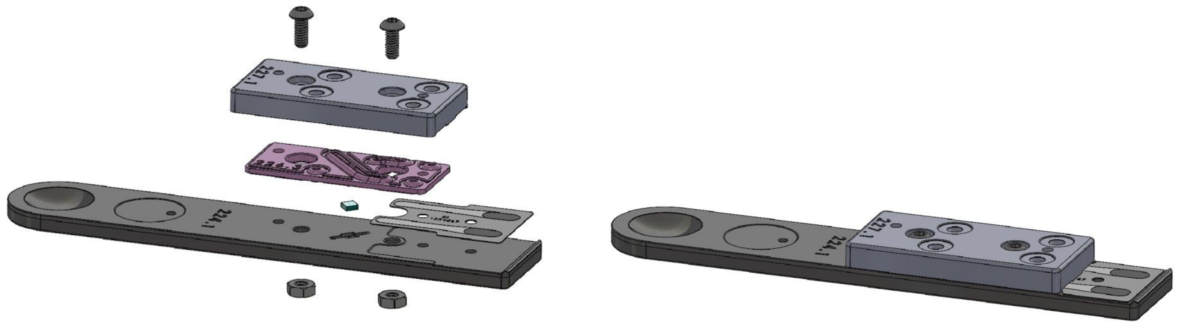

Fig 2. Schematic of the exploded and assembled views of the injection molded test strip. In the exploded view, the small square die between the molded gasket and bottom is the 3mm X 3mm nanopore chip.

References

1. Morin TJ, McKenna WL, Shropshire TD, Wride DA, Deschamps JD, Liu X, et al. A handheld platform for target protein detection and quantification using disposable nanopore strips. Sci Rep. Nature Publishing Group; 2018 Oct 4;8(1):14834.
2. Morin TJ, Shropshire T, Liu X, Briggs K, Huynh C, Tabard-Cossa V, et al. Nanopore-based target sequence detection. Wanunu M, editor. PLoS ONE. 2016 May 5;11(5):e0154426–21.
