# Supplementary material for: Fast and accurate quantification of insertion-site specific transgene levels from raw seed samples using solid-state nanopore technology
Source: PLoS One. 2019 Dec 27;14(12):e0226719. doi: 10.1371/journal.pone.0226719 (PMC6934305; doi:10.1371/journal.pone.0226719)
Supplement: S2 Text — (PDF) [file pone.0226719.s017.pdf]

Supporting Information for
"Fast and Accurate Quantification of Insertion-site Specific Transgene Levels from Raw Seed Samples using Solid-State Nanopore Technology"

Trait vs. Non-Trait Relative Quantification from Nanopore Data

We measured our PCR products using solid-state nanopore sensors, building on our previous development of that technology [1, 2]. Briefly, the translocation of DNA fragments through a nanopore generates a measurable current signature, and trait and non-trait fragments can be discriminated by differences in their respective event signatures. Support vector machines (SVMs) are used for classifying translocation events [3]. First, two control samples (100% trait DNA with 0% non-trait DNA; and 0% trait DNA with 100% non-trait DNA) are run sequentially on a pore, each run collecting a comparable number of events, and the data are combined to train the model. Event signatures are defined as features and include: duration, median amplitude, max amplitude and area. The combined data is divided (70:30) as training:testing data. For the SVM algorithm, a hyper-parameter grid search on the training datasets is used to find the optimal model (optimizing ROC AUC score with 5-fold cross-validation). After the grid search finds the optimal model using the training data, the test data is classified using that model and scored, as shown in Table 1.

Table 1: The trained model is used to classify the testing dataset events and scores the model's accuracy on unseen event data.

Class label	Precision	Recall	F1 score	Event number
0 (non-trait)	0.94	0.95	0.94	269
1 (trait)	0.95	0.95	0.95	306
average	0.95	0.95	0.95	575 (total)

The confusion matrix is also generated from the total of 575 events:

	Predicted non-trait	Predicted trait
Actual non-trait	255	14
Actual trait	16	290

False negative and false positive (FN/FP) rates and model accuracy are derived from this matrix. Next, a control mixture with a known ratio $X{:}Y$ of trait:non-trait molecules (nominally, 1:1) is run on the same pore, and is used to correct for the difference in capture frequency between the trait and non-trait molecules. The equations and mathematical method for using the FN/FP and control mixture are detailed in [4], and summarized here. The estimated fraction of trait molecules in the unknown mixture is denoted F_{mix} and is given by the equation

$$F_{\text{mix}} = \frac{\rho\alpha}{\rho\alpha + 1}, \quad \text{where} \quad \rho = \left(\frac{Q_{\text{mix}} - Q_0}{Q_1 - Q_{\text{mix}}}\right), \quad \alpha = \left(\frac{Q_1 - Q_{X:Y}}{Q_{X:Y} - Q_0}\right) \times \frac{X}{Y}. \tag{1}$$

The four variables $Q_1, Q_0, Q_{X:Y}, Q_{\text{mix}}$ are the fraction of model-identified trait events for each of the four reagents run on the pore: the first three control regent sets (100% trait, 100% non-trait, $X{:}Y$ control mixture), and the unknown mixture. Figure 1 shows representative nanopore event populations from the first two control regent sets (100% trait, 100% non-trait) overlaid along with the model-identified boundary between trait and non-trait events (Fig. 1a), and the results of the model-identified event binning applied to an "unknown" mixture (Fig. 1b) that is 30% trait.

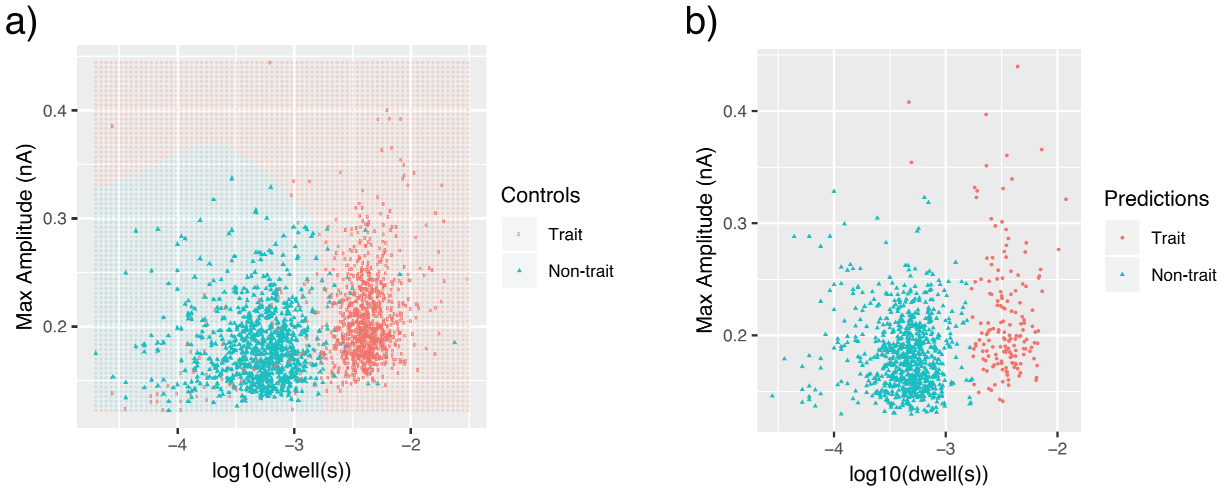

Figure 1: **Nanopore event populations from (a) controls and (b) unknown mixture reagent runs, with model-based boundary for trait vs. non-trait event binning created in (a) and applied in (b).** **(a)** Superposition of events (max amplitude vs. base-10 log of dwell time duration) from 100% trait and 100% non-trait controls that were sequentially recorded, and the identified model-based grid boundary that is subsequently used for predictions. **(b)** Events from unknown reagents after binning each event using the model-based grid boundary in (a). The true mixture is 30% trait, and the SVM prediction after applying equation (1) is 27.7%.

The SVM prediction after applying equation (1) to the data in Figure 1b is 27.7%, compared to the known value of 30% trait. The FN/FP were 6.0% and 3.9% from a total

of 1008 and 907 events recorded, respectively. The control mixture produced $Q_{1:1} = 0.3192$ fraction of model-identified trait events out of 943 total events, while the unknown mixture produced $Q_{\text{mix}} = 0.1717$ fraction of model-identified trait events out of 967 total events.

The results presented in the main text and in S6 Table combined the SVM predictions of four independent nanopore results. Each nanopore runs three controls and one or more (but less than five) mixtures that were treated as unknowns. The combined predictions are the mean of the four predictions generated for a common %Trait value across four independent nanopores.

Since the SVM method requires three controls, we also developed and applied a single-control based method to reduce the number of controls required for prediction. The alternative method uses principle component analysis (PCA) [5], and requires only the control mixture. The method finds the best linear combination of event parameters that maximally divides the control mixture into two subsets on a single axis (Figure 2). The dividing line between the subsets is then used to identify trait vs. non-trait events from the unknown mixture (no FN/FP correction is performed), and a control mixture ratio is still used as a correction. This is equivalent to applying equation (1) and setting $Q_1 = 1$ and $Q_0 = 0$. The PCA prediction for the same data shown in Figure 1b is 30.3%. The raw value for the control mixture was $Q_{1:1} = 0.2916$ model-identified trait events out of 943 total events, while the raw unknown mixture produced $Q_{\text{mix}} = 0.1520$ fraction of model-identified trait events out of 967 total events. The results of applying the PCA method are provided in S6 Table.

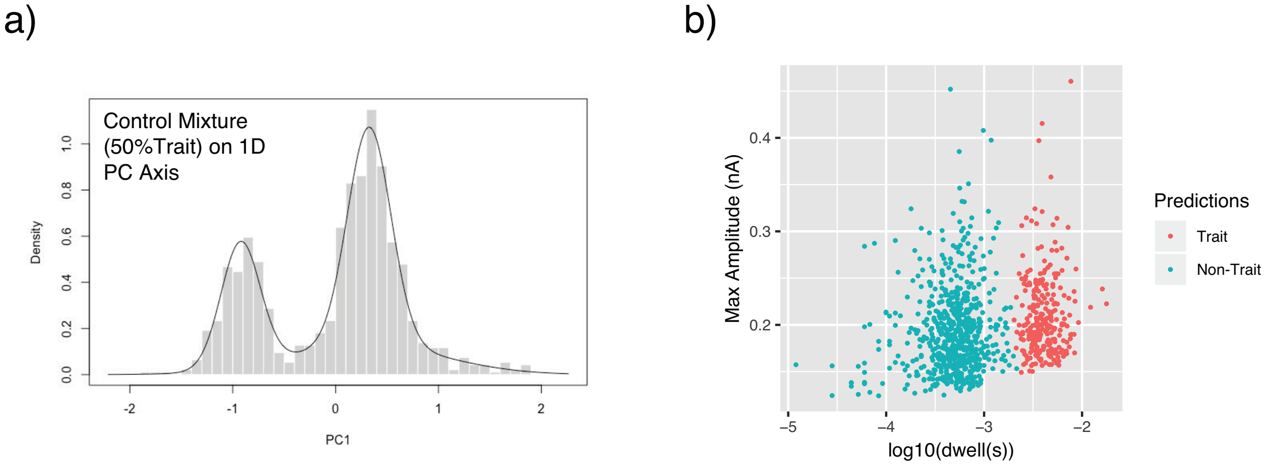

Figure 2: **Principle component analysis uses single 50% trait control mixture and then predicts trait % for the unknown mixture**. **(a)** The clustering result of the 50% mixture based on PCA, projected onto the one dimensional (1D) principal component (PC) axis that maximizes separation in event parameter space. **(b)** Events from the 50% mixture reagent that is shown after PCA in part (a). This is the same control mixture used for the SVM results in Figure 1.

Algorithms were developed and applied using custom code written in R. The SVM method is from library e1071:

`https://www.rdocumentation.org/packages/e1071/versions/1.7-2`

References

[1] Trevor J Morin, William L McKenna, Tyler D Shropshire, Dustin A Wride, Joshua D Deschamps, Xu Liu, Reto Stamm, Hongyun Wang, and William B Dunbar. A handheld platform for target protein detection and quantification using disposable nanopore strips. *Scientific Reports*, 8(1):14834, October 2018.

[2] Trevor J Morin, Tyler Shropshire, Xu Liu, Kyle Briggs, Cindy Huynh, Vincent Tabard-Cossa, Hongyun Wang, and William B Dunbar. Nanopore-based target sequence detection. *PloS ONE*, 11(5):e0154426–21, May 2016.

[3] N Cristianini and J Shawe-Taylor. *An introduction to support vector machines and other kernel-based learning methods.* Cambridge University Press, 2000.

[4] Yanan Zhao, William Mckenna, and William B Dunbar. Fractional abundance of polynucleotide sequences in a sample, International patent no. WO2018081178A1, May 2018.

[5] Ian Jolliffe. *Principal Component Analysis.* Springer, Berlin, Heidelberg, 2011.
